# Supplementary material for: Automatic generation of model sequences for complex regions in assembly graphs with TTT
Source: Cell Genom. 2026 Aug 6;6(8):101323. doi: 10.1016/j.xgen.2026.101323 (PMC13477013; doi:10.1016/j.xgen.2026.101323)
Supplement: Supplementary file 2 [file mmc2.pdf]

Cell Genomics

Transparent Peer Review Record

Summary

Initial submission:	Received : 3/6/2026
	Scientific editors: Judith Nicholson
First round of review:	Number of reviewers: 2<br>Revision invited : 3/30/2026<br>Revision received : 5/21/2026
Second round of review:	Number of reviewers: 2<br>Accepted : 7/9/2026
Data freely available:	Yes
Code freely available:	Yes

This transparent peer review record is not systematically proofread, type-set, or edited. Special characters, formatting, and equations may fail to render properly. Standard procedural text within the editor's letters has been deleted for the sake of brevity, but all official correspondence specific to the manuscript has been preserved.

CellPress

Referees' reports, first round of review

Reviewer #1: Antipov et al. present the Trivial Tangle Traverser (TTT), a tool designed to disentangle complex genomic regions in assembly graphs. Resolving these tangles is arguably one of the most important remaining challenges in the telomere-to-telomere (T2T) assembly era. Although TTT does not solve the problem universally and its application remains somewhat constrained, it establishes a solid algorithmic framework and represents a meaningful step forward for the field. That said, its practical utility in real-world genome assembly projects currently remains obscure; because the generated model sequences are essentially synthetic interpolations, confidently evaluating their true biological quality and accuracy is challenging.

Overall, the manuscript is highly relevant to the genome assembly community and is a strong candidate for publication in Cell Genomics, provided the authors can address the following points.

(1) While reading the paper, I found that there are several prerequisites and constraints for TTT's successful application. Currently, these are scattered throughout the text. The manuscript would greatly benefit from a centralised, explicit statement outlining these operational boundaries so that users can clearly understand the scope and limitations of the tool. For examples,
*** For baseline resolution, the incoming and outgoing anchoring sequences for the tangle must be single-copy.**
*** In cases involving multiple incoming and outgoing boundaries, these anchoring sequences must be explicitly paired by upstream tools (e.g., verkko scaffolding results).**
*** TTT relies heavily on sequence coverage estimates generated by the upstream assembler - but not all assemblers provide highly accurate coverage estimates, particularly for the highly repetitive sequences found within these tangles, which may only contain a few simple variants.**

(2) Regarding the evaluation of TTT, it would be beneficial to disclose more information and expand the discussion on the following points:
*** The complexity of the tangles TTT can handle is not clear. It would be good to provide more information of the tangles that were successfully resolved in the T2T-HG002, such as the number of sequences per tangle, average resulting edge multiplicities, and average node degrees, etc.**
*** The authors note that if no valid Eulerian path exists that covers all sequences, TTT drops "the requirement that all reasonably covered edges are used to ensure that some solution will be found". An explicit discussion quantifying how frequently this fallback is triggered in the tested datasets should be included. When it does occur, how many "reasonably covered" sequences are typically excluded from the final model?**
*** In the dual-path scenario, TTT introduces an auxiliary edge to bridge the two paths. However, when this auxiliary edge is visited can sometimes be quite arbitrary due to the repetitive nature of the tangled sequences, potentially leading to quite imbalanced haplotypes or misplacement of sequences. Optimisation based on long-read alignment scores may not fully resolve this issue, as most subpaths will likely score similarly.**

(3) In the Discussion, the authors state "Nonetheless, TTT's algorithm is designed to ensure that every edge of reasonable coverage is included in the final traversal, meaning all repeat variations will be present..." This statement appears to contradict the Methods section, which describes a fallback mechanism where the requirement to use all reasonably covered edges is explicitly removed if no Eulerian path can be found.

Reviewer #2: The manuscript presents Trivial Tangle Traverser (TTT), a method for resolving complex tangles in assembly graphs by combining coverage-based edge multiplicity estimation and read-to-graph alignment-supported traversal optimization. The method is clearly described, and the zebra finch chrZ application provides an example of practical utility. However, the current manuscript still requires substantial clarification regarding method scope, benchmarking, and validation in several important scenarios. In particular, author did not compare TTT with hifiasm on tangle resolving, and the applicability boundaries of TTT seems narrow, the comparison with verkko does not support superiority claims, and validation for haplotype-resolved assemblies remains insufficient.
Major comments

1. Method scope should be defined more explicitly. TTT requires unique, non-repetitive, non-homozygous boundary edges. This restricts its applicability to a subset of residual tangles and excludes more general repeat structures. This limitation should be stated more prominently throughout the manuscript.
2. The assumptions underlying multiplicity estimation require stronger discussion. The MILP formulation is a strength of the method, but it depends on local coverage uniformity across genomic paths traversing the tangle. The manuscript clearly states that TTT depends on several strong assumptions, most notably the presence of unique/nonrepetitive/non-homozygous boundary edges and approximate local coverage uniformity across genomic paths traversing the tangle. However, the current study does not provide a systematic evaluation of how often these assumptions are satisfied in real residual tangles from biological genomes. The HG002 and zebra finch analyses demonstrate applicability in selected cases, but they do not establish the prevalence of TTT-compatible tangles, nor do they quantify how sensitive the method is to violations of these assumptions. I therefore encourage the authors to add a dedicated analysis of assumption validity in real datasets, for example by reporting the fraction of candidate tangles with identifiable valid boundary edges, measuring local coverage uniformity across residual tangles, and stratifying TTT performance by degree of assumption satisfaction. Without such analysis, it remains difficult to assess the practical scope and generalizability of the method.
3. The traversal optimization step is heuristic and should be described as such more rigorously. The swap-based optimization is a local search procedure initialized from random Eulerian paths. Although practical, it does not guarantee a globally optimal traversal. The manuscript should avoid any wording that could be interpreted as implying theoretical optimality.
4. The comparison with Hifiasm was not included. Hifiasm, particularly Hifiasm (ONT), is one of the most popular T2T or near-T2T assemblers. How does TTT compare to the tangle-resolving solutions in Hifiasm?
5. The comparison with verkko does not demonstrate superiority. In HG002, most paths are concordant between TTT and verkko, but in discrepant cases verkko outperforms TTT more often than the reverse. In addition, the benchmark is restricted to tangles already resolved by verkko, which biases the analysis toward easier cases. The manuscript should frame this result strictly as a consistency check rather than a superiority benchmark.
6. Validation in haplotype-resolved settings is insufficient. Although the manuscript discusses handling of two-haplotype tangles, it does not provide dedicated evaluation of phasing-related accuracy, such as switch error or local phasing consistency. This is an important omission for diploid or haplotype-resolved assemblies.
7. Important non-applicable cases should be emphasized more clearly. The limitations of TTT should be stated more prominently and explicitly in the main text. In the Discussion, the authors note that TTT cannot handle inter-chromosomal repeats, such as human rDNA-associated tangles, and is not intended for gaps caused primarily by insufficient coverage. These are not minor edge cases. On the contrary, such regions represent some of the most difficult unresolved problems in current genome assembly, including for state-ofthe-art assemblers. In practice, large multi-chromosomal repeat structures and coveragelimited unresolved regions are precisely the types of assembly failures that remain hardest to resolve automatically. Because TTT requires unique, non-repetitive boundary edges and relies on local coverage-based multiplicity estimation, it is inherently not applicable to many of these biologically important and technically challenging regions. I therefore recommend that the authors summarize these non-applicable cases much more clearly in the Abstract, Introduction, and main Results/Discussion text, so that readers can accurately understand that TTT is a method for a specific subset of residual tangles.
8. The validation scale remains limited. The manuscript relies mainly on HG002 and zebra finch chrZ. Additional testing across more species, repeat architectures, and sequencing conditions would strengthen confidence in the generality of the method.

Minor comments
1. There appears to be a missing punctuation mark in the zebra finch Results section. In the sentence describing the two unresolved tangles, the text reads: "The first tangle contained 45 edges, and the second tangle contained 32 edges In both cases, …". A period should be inserted after "32 edges".
3. One sentence in the Figure 3-related Results text appears to be missing a final period. The sentence "Of the 200 copies, only 4 of them present frameshift issues, suggesting that they may largely be functional" should end with a period. The phrasing could also be slightly improved for clarity, for example by revising "present frameshift issues" to "show frameshift defects/issues".
4. There is an spelling error in the Supplemental Methods. The term "reverse_compement(edge)" appears in the supplementary text, whereas the correct spelling elsewhere is

"reverse_complement(edge)". The authors should check and correct this inconsistency.
5. The statement that two runs crashed should be clarified. The manuscript states that "Two runs crashed, 218 succeeded", but does not explain the cause of these crashes, whether they were associated with specific graph structures, or whether they have any implications for interpretation of the benchmark. A brief clarification in the main text or supplementary materials would be helpful.

Authors' response to the first round of review

We thank Dr. Nicholson and the reviewers for their detailed reviews and helpful comments for our manuscript. Here we provide a point-by-point response, with the original reviewer comments in black and our responses in blue.

Reviewer 1.

(1) While reading the paper, I found that there are several prerequisites and constraints for TTT's successful application. Currently, these are scattered throughout the text. The manuscript would greatly benefit from a centralised, explicit statement outlining these operational boundaries so that users can clearly understand the scope and limitations of the tool. For examples,

* For baseline resolution, the incoming and outgoing anchoring sequences for the tangle must be single-copy.
* In cases involving multiple incoming and outgoing boundaries, these anchoring sequences must be explicitly paired by upstream tools (e.g., verkko scaffolding results).
* TTT relies heavily on sequence coverage estimates generated by the upstream assembler - but not all assemblers provide highly accurate coverage estimates, particularly for the highly repetitive sequences found within these tangles, which may only contain a few simple variants.

Thank you for this constructive feedback. We have combined all prerequisites and constraints for TTT's successful application into two dedicated subsections within the Methods and Discussion sections.

(2) Regarding the evaluation of TTT, it would be beneficial to disclose more information and expand the discussion on the following points:

* The complexity of the tangles TTT can handle is not clear. It would be good to provide more information of the tangles that were successfully resolved in the T2T-HG002, such as the number of sequences per tangle, average resulting edge multiplicities, and average node degrees, etc.

Thank you. We have summarized the requested complexity metrics of the tangles TTT can handle in Supplemental Tables S3 and S4. We also noted that benchmark T2T-HG002 tangles are, on average, significantly simpler than real world cases that Verkko was not able to handle directly (e.g., zebra finch chrZ), as indicated by the median number of edges (7 edges in T2T-HG002 compared to 32 and 45 edges in the zebra finch tangles). Complexity of the tangles in the new giraffe benchmarking dataset is somewhere in between with median 17 edges for all TTT-resolved tangles and 9 edges for TTT-resolved tangles with a verkko traversal available for comparison.

* The authors note that if no valid Eulerian path exists that covers all sequences, TTT drops "the requirement that all reasonably covered edges are used to ensure that some solution will be found". An explicit discussion quantifying how frequently this fallback is triggered in the tested datasets should be included. When it does occur, how many "reasonably covered" sequences are typically excluded from the final model?

Response to Reviewers

This rollback didn't happen in any of 246 tangles used for benchmarking. We've clarified this in

the text.
* In the dual-path scenario, TTT introduces an auxiliary edge to bridge the two paths. However, when this auxiliary edge is visited can sometimes be quite arbitrary due to the repetitive nature of the tangled sequences, potentially leading to quite imbalanced haplotypes or misplacement of sequences. Optimisation based on long-read alignment scores may not fully resolve this issue, as most subpaths will likely score similarly.
We agree this is a valid concern. To address it, we have introduced a small penalty during path scoring for cases where all copies of a node (with multiplicity > 1) are assigned to a single haplotype. We acknowledge, however, that accurately determining the haplotype location of a large duplication is inherently challenging and likely requires additional information such as trio or Hi-C data. We also added this limitation to the Discussion section.
"A common scenario in diploid tangles involves sequences present at different copy numbers between haplotypes, for example due to a duplication event in only one haplotype. Failure of the assembler to resolve such regions typically indicates ambiguity in assigning repeat copies to haplotypes or in estimating haplotype-specific copy numbers. Accordingly, while TTT produces traversals consistent with read alignments – and, in ambiguous cases, ensures that each haplotype contains at least one copy of a repeat – caution should be exercised when using these traversals to infer haplotype-specific copy number or exact per-haplotype duplication status."
(3) In the Discussion, the authors state "Nonetheless, TTT's algorithm is designed to ensure that every edge of reasonable coverage is included in the final traversal, meaning all repeat variations will be present..." This statement appears to contradict the Methods section, which describes a fallback mechanism where the requirement to use all reasonably covered edges is explicitly removed if no Eulerian path can be found.
While noting that the fallback mechanism never occurred in the benchmark, we have reworded the corresponding phrase in the Discussion to clarify that including every edge is not always possible, particularly due to graph construction or coverage errors.
"TTT's algorithm is designed to ensure that, with exception of clearly contradictive assembly graph and coverage, every edge of reasonable coverage is included in the final traversal,"

Reviewer 2:

1. Method scope should be defined more explicitly. TTT requires unique, non-repetitive, non-homozygous boundary edges. This restricts its applicability to a subset of residual tangles and excludes more general repeat structures. This limitation should be stated more prominently throughout the manuscript.
We appreciate the opportunity to clarify our terminology, as the misunderstanding stems from the different meanings of 'repetitive/unique.' We use 'non-repetitive' in the context of the assembly graph, meaning the edge should occur only once in the final haplotype-resolved genome assembly. While it is possible for all edges between a gap and the scaffold end to be non-unique, this is rare for T2T assemblies. Our evaluation of 227 HPRC T2T assemblies found this occurs for less than 6% of gaps (see also new limitation subsection and Supplemental Table S2). We have added an explicit clarification that our graph-based uniqueness definition differs from a sequence content-based definition.
"TTT imposes an additional requirement for the tangles it resolves: each boundary edge x_i and y_i should be unique – i.e., non-repetitive and non-homozygous edges that should be present only once in a correct genome assembly. It is important to note that this graph-based definition is different from sequence-based definitions. For example a

diverged copy of a repeat is typically represented by a separate edge in the assembly graph and thus is considered unique. "

2. The assumptions underlying multiplicity estimation require stronger discussion. The MILP formulation is a strength of the method, but it depends on local coverage uniformity across genomic paths traversing the tangle. The manuscript clearly states that TTT depends on several strong assumptions, most notably the presence of unique/nonrepetitive/non-homozygous boundary edges and approximate local coverage uniformity across genomic paths traversing the tangle. However, the current study does not provide a systematic evaluation of how often these assumptions are satisfied in real residual tangles from biological genomes. The HG002 and zebra finch analyses demonstrate applicability in selected cases, but they do not establish the prevalence of TTT-compatible tangles, nor do they quantify how sensitive the method is to violations of these assumptions. I therefore encourage the authors to add a dedicated analysis of assumption validity in real datasets, for example by reporting the fraction of candidate tangles with identifiable valid boundary edges, measuring local coverage uniformity across residual tangles, and stratifying TTT performance by degree of assumption satisfaction. Without such analysis, it remains difficult to assess the practical scope and generalizability of the method.

We thank the reviewer for encouraging this analysis. We have added a separate dedicated subsection on TTT limitations. In this section, we classified all gaps in hundreds of HPRC T2T assemblies into TTT-applicable and TTT-inapplicable categories, finding approximately half of them to be TTT-applicable (Supplemental Table S2). Regarding local coverage uniformity, since the ground truth is unknown, a full evaluation for that datasets is not possible. However, Supplemental Figure S1 demonstrates that coverage inconsistency is generally low for tangles in the T2T-HG002 dataset. We provide a coverage inconsistency measure within TTT as guidance, but leave the final decision to use a TTT generated traversal to the user.

3. The traversal optimization step is heuristic and should be described as such more rigorously. The swap-based optimization is a local search procedure initialized from random Eulerian paths. Although practical, it does not guarantee a globally optimal traversal. The manuscript should avoid any wording that could be interpreted as implying theoretical optimality.

We agree entirely. We have clarified that both the traversal optimization and the subsequent Mixed-Integer Linear Programming (MILP) step are heuristic and do not guarantee theoretical optimality of the solution. We have added clarifications to the abstract, introduction, methods and discussion to rigorously distinguish between the optimization problem formulation and the provided solution.

"Although the goal is to find a path that is most consistent with the sequencing data, TTT's output may be a local and not a global optimum because of the heuristic nature of the algorithm."

4. The comparison with hifiasm was not included. Hifiasm, particularly Hifiasm (ONT), is one of the most popular T2T or near-T2T assemblers. How does TTT compare to the tangle-resolving solutions in Hifiasm?

We want to emphasize that TTT is not a competitor to traditional assemblers like hifiasm or verkko. The primary goal of TTT is to provide a plausible solution for cases where genome assemblers fail to yield a result. Therefore, there is no way to directly compare against hifiasm and verkko because TTT's added value is precisely for the tangles they will not resolve. That said, we tested hifiasm on the zebra finch test case. Hifiasm was unable to scaffold or traverse either of the target gaps, and we have added this result to the manuscript. Our comparison with verkko on the T2T-HG002 dataset serves as a consistency check to demonstrate only that TTT behaves well for simple, verifiable cases and is not a "bakeoff". In response to another comment below, we have added an additional validation experiment for the giraffe genome to further

demonstrate the reasonableness of TTT output on verifiable cases.

5. The comparison with verkko does not demonstrate superiority. In HG002, most paths are concordant between TTT and verkko, but in discrepant cases verkko outperforms TTT more often than the reverse. In addition, the benchmark is restricted to tangles already resolved by verkko, which biases the analysis toward easier cases. The manuscript should frame this result strictly as a consistency check rather than a superiority benchmark.

Superiority benchmarking was not our intent. We have added additional clarification to the text emphasizing that this experiment is a consistency check on easily verifiable cases.

“This test focused only on those tangles where a verkko resolution was available and therefore is biased towards simpler, resolvable tangles (see Supplemental table S3 for the tangle complexity statistics). We do not expect TTT to outperform a state of the art T2T genome assembler that is able to use more data (coverage information from different technologies and Hi-C data). While this test does not demonstrate an improvement over verkko’s resolution, the fact that for most of the tangles TTT’s results match verkko’s confirms that TTT is consistent and comparable with existing resolution methods and serves as a sanity check.”

6. Validation in haplotype-resolved settings is insufficient. Although the manuscript discusses handling of two-haplotype tangles, it does not provide dedicated evaluation of phasing-related accuracy, such as switch error or local phasing consistency. This is an important omission for diploid or haplotype-resolved assemblies.

A total of 179 of 218 (82%) TTT-HG002 tangles were diploid, and we demonstrate that TTT's performance on the majority of these tangles is equivalent to a modern diploid genome assembler (verkko), yielding exactly the same local phasing consistency. We have expanded the Discussion to further address the diploid case:

“In our benchmark, a total of 179 of 218 (82%) T2T-HG002 tangles were diploid, and TTT's performance on 163 (91%) of these diploid tangles is equivalent to a modern diploid genome assembler (verkko), yielding exactly the same local phasing consistency. However, for more complex diploid tangles which were unresolved by a T2T assembler, a common scenario involves sequences present at different copy numbers between haplotypes, for example due to a duplication event in only one haplotype. Failure of the assembler to resolve such regions typically indicates ambiguity in assigning repeat copies to haplotypes or in estimating haplotype-specific copy numbers. Accordingly, while TTT produces traversals consistent with read alignments – and, in ambiguous cases, ensures that each haplotype contains at least one copy of a repeat – caution should be exercised when using these traversals to infer haplotype-specific copy number or exact per-haplotype duplication status.”

7. Important non-applicable cases should be emphasized more clearly. The limitations of TTT should be stated more prominently and explicitly in the main text. In the Discussion, the authors note that TTT cannot handle inter-chromosomal repeats, such as human rDNA-associated tangles, and is not intended for gaps caused primarily by insufficient coverage. These are not minor edge cases. On the contrary, such regions represent some of the most difficult unresolved problems in current genome assembly, including for state-of-the-art assemblers. In practice, large multi-chromosomal repeat structures and coveragelimited unresolved regions are precisely the types of assembly failures that remain hardest to resolve automatically. Because TTT requires unique, non-repetitive boundary edges and relies on local coverage-based multiplicity estimation, it is inherently not applicable to many of these biologically important and technically challenging regions. I therefore recommend that the authors summarize these non-applicable cases much more clearly in the Abstract, Introduction, and main

Results/Discussion text, so that readers can accurately understand that TTT is a method for a specific subset of residual tangles.

We recognize the importance of clearly stating TTT's limitations. We have added prerequisites and constraints for TTT's application to the Methods ("Requirements") and Discussion ("Limitations of the study") sections. We believe this addresses the reviewer's request without requiring repetition in the Abstract and Introduction.

8. The validation scale remains limited. The manuscript relies mainly on HG002 and zebra finch chrZ. Additional testing across more species, repeat architectures, and sequencing conditions would strengthen confidence in the generality of the method.

To strengthen our evaluation, we have added a dedicated subsection demonstrating TTT performance on the recently published giraffe T2T dataset which also showed that TTT's results are consistent on verifiable tangles from another species. Variability in sequencing coverage within tangles is still possible and could affect TTT results, as coverage fluctuates with sequencer type, chemistry updates, and library preparation methods (see response to question 2 above). Overall, a comprehensive evaluation of this effect was beyond the scope of this study, which aimed at reporting a novel method to enable for the first time the study of some of the most complex regions of genomes, recalcitrant even to T2T assembly, and the important biological findings that can be derived from these sequence models. Yet, we have tested TTT on human, ruminant, and bird genomes showing consistent performance, which is comparable to the variety of species used for validation in other recent assembly papers (e.g. hifiasm, verkko). We feel this captures a wide range of real assembly graph cases, confirming TTT's applicability. The manuscript was revised to better capture these aspects.

Minor comments

Thank you for noticing those issues

1. There appears to be a missing punctuation mark in the zebra finch Results section. In the sentence describing the two unresolved tangles, the text reads: "The first tangle contained 45 edges, and the second tangle contained 32 edges In both cases, …". A period should be inserted after "32 edges".

Fixed

3. One sentence in the Figure 3-related Results text appears to be missing a final period. The sentence "Of the 200 copies, only 4 of them present frameshift issues, suggesting that they may largely be functional" should end with a period. The phrasing could also be slightly improved for clarity, for example by revising "present frameshift issues" to "show frameshift defects/issues".

Fixed

4. There is an spelling error in the Supplemental Methods. The term "reverse_compement(edge)" appears in the supplementary text, whereas the correct spelling elsewhere is "reverse_complement(edge)". The authors should check and correct this inconsistency.

Fixed

5. The statement that two runs crashed should be clarified. The manuscript states that "Two runs crashed, 218 succeeded", but does not explain the cause of these crashes, whether they were associated with specific graph structures, or whether they have any implications for interpretation of the benchmark. A brief clarification in the main text or supplementary materials would be helpful.

Both crashes happened because of the errors of tangle detection scripts (two telomeric regions from the same arm of a chromosome were erroneously considered as incoming and outgoing edges). This is now mentioned in the text.

Referees' reports, second round of review

Reviewer #1: The authors have well addressed my previous concerns. The additional analyses and clarifications substantially strengthened the manuscript. I only have two minor comments. I support publication after minor revision.

1. In the T2T-giraffe dataset section, the authors wrote "... TTT's solutions are unlikely to be substantially incorrect". I feel the statement is somewhat stronger than what the results suggested: TTT resolved 38 gaps, but 19 of these could not be validated as verkko failed; for the remaining 19 gaps, TTT produced results differed from verkko in three cases. While the results are encouraging, I suggest softening the sentence. Also, it would be useful to provide more information about the three cases where two tools differed, especially the read mapping results - which solutions are better supported?

2. In the Methods the authors reported some results on the HPRC human assemblies, such as in the "TTT algorithm" section "...this situation is uncommon in near-T2T assemblies: among 8,532 gaps across 227 assemblies..." and in the "Requirements" section "We quantified the proportion of TTT-applicable gaps across 227 HPRC human assemblies...". It would be good to move them to the Results or Discussion section, or at least to reiterate these figures, so readers can easily find them.

Reviewer #2: The authors have addressed most of my concerns. Thank you. However, I still have a minor concern regarding the usage of TTT in the community.
The manuscript would also benefit from clearer documentation of the practical end-to-end workflow. I recommend that the authors provide a more detailed README in the GitHub repository (https://github.com/marbl/TTT). Specifically, the README should include instructions on how to use TTT together with mainstream assemblers-not only Verkko but also HiFiasm. Furthermore, it should describe the content of each output file and explain how these outputs can be combined with the results from Verkko or HiFiasm to improve genome assembly quality.

Authors' response to the second round of review

Reviewer #1: The authors have well addressed my previous concerns. The additional analyses and clarifications substantially strengthened the manuscript. I only have two minor comments. I support publication after minor revision.
1. In the T2T-giraffe dataset section, the authors wrote "... TTT's solutions are unlikely to be substantially incorrect". I feel the statement is somewhat stronger than what the results suggested: TTT resolved 38 gaps, but 19 of these could not be validated as verkko failed; for the remaining 19 gaps, TTT produced results differed from verkko in three cases. While the results are encouraging, I suggest softening the sentence. Also, it would be useful to provide more information about the three cases where two tools differed, especially the read mapping results - which solutions are better supported?
Thank you! We rephrased the corresponding sentence.
These results indicate that TTT produces traversals consistent with a state-of-the-art genome assembler when both approaches yield solutions, and provide some confidence in TTT's solutions in cases lacking assembler-derived ground truth, although their accuracy cannot be directly assessed.

In the three cases where the tools differ, we do not see significant differences in read mapping. Manual examination suggests that in two cases verkko was correct and TTT was suboptimal because of the uneven coverage within a tangle. In the third case, verkko's traversal didn't include some reasonably covered nodes from the initial graph, suggesting that the TTT model was better. However, without a clear quality metric we prefer not to include such vague conclusions into the paper text.

2. In the Methods the authors reported some results on the HPRC human assemblies, such as in the "TTT algorithm" section "...this situation is uncommon in near-T2T assemblies: among 8,532 gaps across 227 assemblies..." and in the "Requirements" section "We quantified the proportion of TTT-applicable gaps across 227 HPRC human assemblies...". It would be good to move them to the Results or Discussion section, or at least to reiterate these figures, so readers can easily find them.

Thank you, we added directly reference on those results in Discussion

TTT is not universal and cannot be applied to every gap in an assembly. Detailed requirements are listed in the methods section. Across 8,532 gaps in 227 HPRC human assemblies generated with Verkko v2.3 48.9% gaps were TTT-applicable (see also Supplementary table S2).

Response to Reviewers

Reviewer #2: The authors have addressed most of my concerns. Thank you. However, I still have a minor concern regarding the usage of TTT in the community.

The manuscript would also benefit from clearer documentation of the practical end-to-end workflow. I recommend that the authors provide a more detailed README in the GitHub repository (https://github.com/marbl/TTT). Specifically, the README should include instructions on how to use TTT together with mainstream assemblers-not only Verkko but also HiFiasm. Furthermore, it should describe the content of each output file and explain how these outputs can be combined with the results from Verkko or HiFiasm to improve genome assembly quality.

Thank you, we've addressed those issues in the README on github
