# Supplementary material for: Automatic generation of model sequences for complex regions in assembly graphs with TTT
Source: Cell Genom. 2026 Aug 6;6(8):101323. doi: 10.1016/j.xgen.2026.101323 (PMC13477013; doi:10.1016/j.xgen.2026.101323)
Supplement: Supplementary file 3 [file mmc3.pdf]

Cell Genomics

Short article

Automatic generation of model sequences for complex regions in assembly graphs with TTT

Graphical abstract

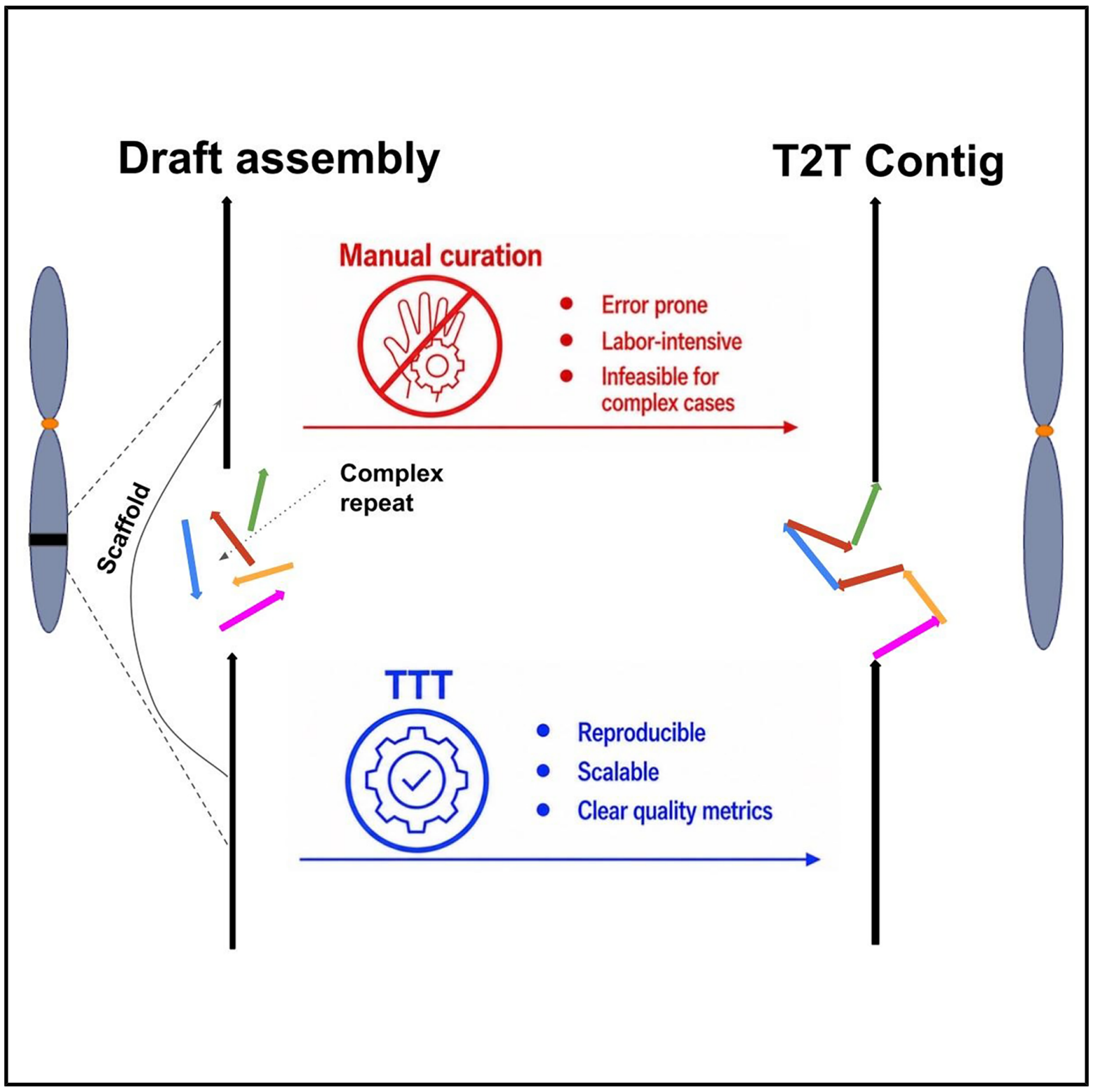

Authors

Dmitry Antipov, Ying Chen, Marco Sollitto, Adam M. Phillippy, Giulio Formenti, Sergey Koren

Correspondence

aphillippy@jhu.edu (A.M.P.), gformenti@rockefeller.edu (G.F.), sergekoren@gmail.com (S.K.)

In brief

Antipov et al. present a tool that automates gap closure in complex genomic regions. This method replaces error-prone, labor-intensive manual curation with heuristic optimization of objective quality metrics, enabling efficient modeling of complex regions such as multi-megabase gene arrays for T2T genome assembly projects.

Highlights

- Genome assemblers favor correctness, leaving gaps in complex genome regions
- T2T projects often use manual, error-prone curation to bridge assembly gaps
- Our method replaces manual curation by optimizing objective quality metrics
- We validated our method and applied it to traverse multi-megabase gene arrays

Antipov et al., 2026, Cell Genomics 6, 101323
August 12, 2026 Published by Elsevier Inc.
https://doi.org/10.1016/j.xgen.2026.101323

Cell Genomics

CellPress
OPEN ACCESS

Short article

Automatic generation of model sequences for complex regions in assembly graphs with TTT

Dmitry Antipov,[1] Ying Chen,[2] Marco Sollitto,[2,3] Adam M. Phillippy,[1,4,5,6,*] Giulio Formenti,[2,*] and Sergey Koren[1,7,*]
[1]Genome Informatics Section, Center for Genomics and Data Science Research, National Human Genome Research Institute, National Institute of Health, Bethesda, MD 20892, USA
[2]The Vertebrate Genome Laboratory, The Rockefeller University, New York, NY 10065, USA
[3]Department of Biology, University of Florence, 50019 Sesto Fiorentino, Italy
[4]Department of Computer Science, Johns Hopkins University, Baltimore, MD 21218, USA
[5]Department of Biomedical Engineering, Johns Hopkins University, Baltimore, MD 21218, USA
[6]Department of Genetic Medicine, Johns Hopkins University School of Medicine, Baltimore, MD 21205, USA
[7]Lead contact
*Correspondence: aphillippy@jhu.edu (A.M.P.), gformenti@rockefeller.edu (G.F.), sergekoren@gmail.com (S.K.)
https://doi.org/10.1016/j.xgen.2026.101323

SUMMARY

Recent developments have enabled the automated assembly of vertebrate chromosomes from telomere to telomere. However, for long, highly similar repeats, genome assemblers may leave tangles in the assembly graph and gaps in the assembly. In recently published genomes, such gaps are closed by manual graph curation, a process that is labor intensive, error prone, and sometimes infeasible. Consequently, important genomic regions may be misassembled or omitted. Here, we present the trivial tangle traverser (TTT) algorithm that finds optimized resolutions of assembly graph tangles. TTT uses depth of coverage and read-to-graph alignment information in a two-stage process to estimate sequence multiplicities and identify traversals that are consistent with the underlying data. We evaluate TTT traversals on the HG002 human reference genome, compare TTT with a state-of-the-art assembler on the giraffe T2T assembly, and demonstrate its use to characterize a previously unassembled amplified p21-activated serine/threonine kinase 3-like (*PAK3L*) gene array in the zebra finch genome.

INTRODUCTION

Advances in sequencing technologies have enabled a new goal for *de novo* genome assembly: the complete assembly of entire chromosomes from "telomere to telomere" (T2T). In the T2T-CHM13 project,[1] assembly continuity was achieved by combining previous-era genome assemblers along with specialized semi-manual processing. Subsequently, specific tools focusing on automated T2T genome assembly were developed.[2–6] However, despite all the progress in sequencing technologies and assembly algorithms, some complex genomic regions are still unresolvable with current read length and error profiles.[7,8]

T2T assembly projects benefit from either parental[3,9] or long-range[2,6,10] sequencing data for haplotype separation and scaffolding. Thus, T2T genome assemblers can "jump over" complex genomic regions, leaving gaps between contigs on a scaffold, rather than just stopping at those regions. This can lead to significant differences between the number of T2T contigs and scaffolds in the assembler's output. For example, the Verkko2 paper reported a median count of 21 T2T contigs versus 39 T2T scaffolds across 17 Human Pangenome Reference Consortium (HPRC) human genome assemblies.[6]

T2T assembly projects[1,11–14] aim to resolve gaps and close them by postprocessing the results of the assembler software. Most commonly used gap-closing tools, such as LR_Gapcloser, TGS-GapCloser, and SAMBA,[15–17] rely on finding individual reads that bridge the gap. This limits their usefulness to closing relatively short gaps (100–200 kbp). DEGAP[18] can fill larger gaps through an iterative extension strategy, stopping when no extension is available or when there is sufficient similarity between the extension and the gap boundary. However, these tools can leave gaps in complex regions, such as large repetitive arrays and segmental duplications.[19] Worse, this can lead to gaps being filled with incorrect sequences that can escape validation checks due to read mapping ambiguity in repetitive regions[20] and may go unnoticed by future users of the reference assembly. T2T projects therefore often rely on semi-manual inspection of the assembly graph followed by manual path selection using project-specific scripts.[1,21] The benefit of this approach is that it generally produces a resolution that is consistent with the underlying sequencing data (represented by the assembly graph), but this process requires domain expertise, is labor-intensive, and lacks the reproducibility of automated tools.

Here, we present trivial tangle traverser (TTT), a tool that replaces the error-prone manual gap-closing process with the identification of an optimal tangle traversal in the assembly graph, which then leads to the automated construction of a patch sequence. The problem of approximate gap closure is

This is an open access article under the CC BY license (http://creativecommons.org/licenses/by/4.0/).

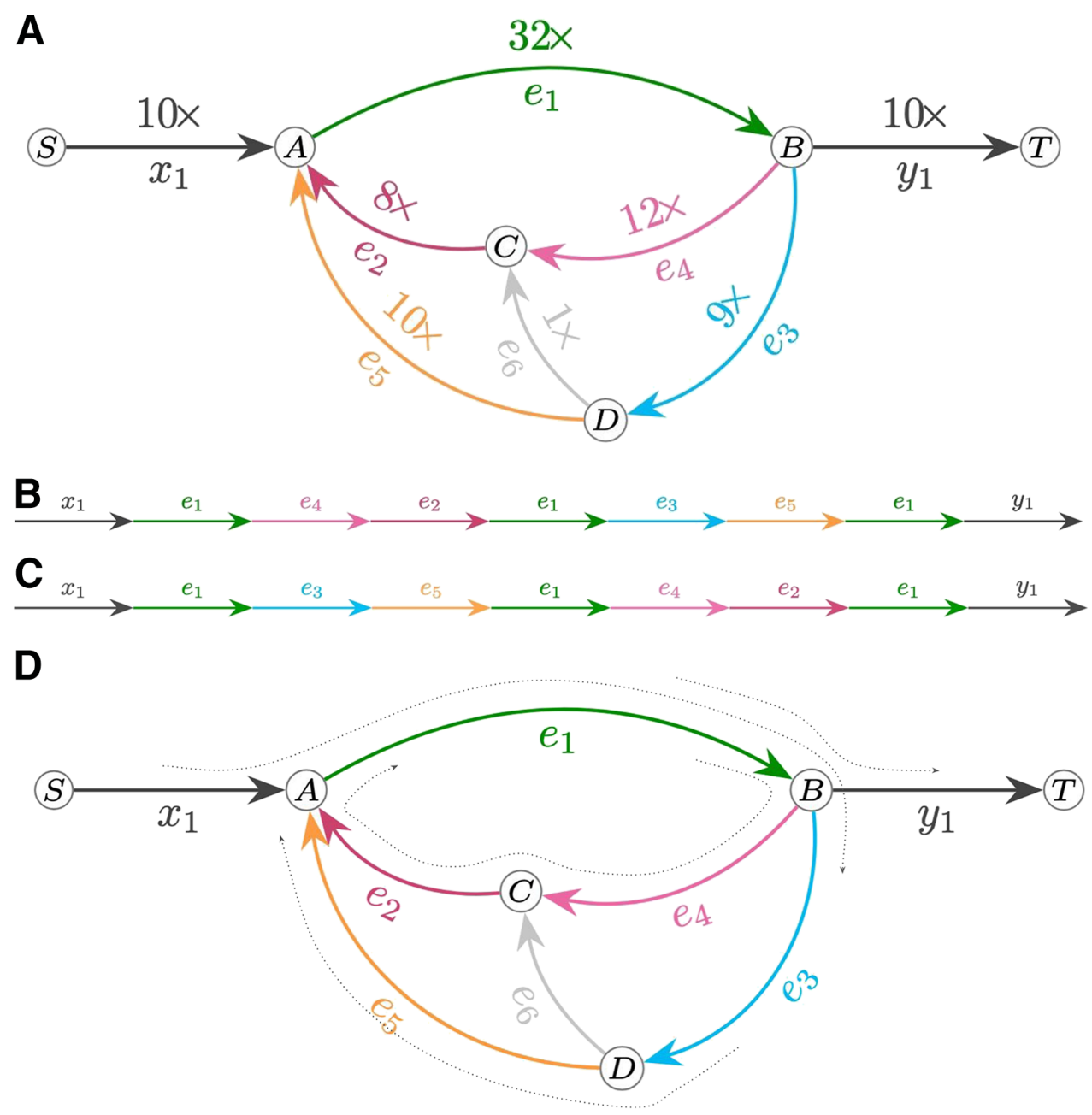

Figure 1. Example of assembly graph tangle traversals

(A) An assembly graph tangle with boundary edges x_1 and y_1 and tangle edges e_1, e_2, … e_6, additionally labeled with coverage information. Capital letters A, B, C, D, S, and T represent the graph vertices.

(B and C) Two possible underlying genomic traversals consistent with the coverage data.

(D) Read alignments to the same graph (dotted curves) demonstrate that the traversal in (C) has better support. Table S1 shows a comparison of Verkko versus TTT on real tangles in HG002. Tables S3 and S4 show statistics of real tangles identified in the HG002 and giraffe assemblies.

different from a typical local or global *de novo* genome assembly problem. Most genome assembly tools prioritize correctness and thus usually stop in ambiguous regions, providing a gapped or fragmented assembly. In complex regions, these highly fragmented or incomplete sequence assemblies can lead to missing gene duplications, hindering analysis. To reveal these dark regions, TTT prioritizes completeness and aims to find a traversal that is most consistent with the input data. Instead of providing a partial solution (i.e., a set of fragmented contigs), it always produces a single continuous path through the tangle. In the case of multiple equally good solutions (e.g., in the case of an exact repeat larger than read length), it will select one of the paths even if its correctness cannot be unambiguously verified. Given current sequencing read lengths and accuracies, there can be multiple such regions in large, complex genomes that are essentially unsolvable. Thus, we refer to the output of our tangle traversals as "model sequences" rather than "assemblies" to communicate this uncertainty. It should be noted that although the goal is to find a path that is most consistent with the sequencing data, TTT's output may be a local, not a global, optimum because of the heuristic nature of the algorithm.

Below, we describe the algorithm in detail, compare TTT with a general-purpose assembler on a large human genome reference test set, and highlight its performance on two previously unassembled megabase-scale gaps on chromosome Z (chrZ) of the zebra finch genome, enabling the study of this complex ampliconic gene array.

RESULTS

To close assembly gaps of arbitrary size, we have implemented a method named the TTT. TTT takes as input an assembly graph, coverage information, and read alignments and searches for a path through the corresponding region of the assembly graph that has the highest support from sequencing coverage and read alignments. Figure 1 shows a simplified example of TTT's input and output. TTT relies on an assumption of sequencing coverage uniformity to estimate the multiplicities of graph edges within a tangle. Since each gap is considered separately, uniformity conditions are required only for local regions and not across the whole genome, thereby mitigating the effects of localized sequencing coverage biases, such as within tandem satellite arrays. TTT reduces the multiplicity estimation problem to a mixed-integer linear programming problem,[22,23] a well-studied class of optimization problems that can be addressed using a variety of highly optimized heuristic solvers. Then, with the determined edge multiplicities and corresponding multigraph, TTT searches for an Eulerian path with the best support from read-to-graph alignments through a gradient-descent-like optimization. Further details on the TTT algorithm are provided in STAR Methods and Methods S1.

Reference-based evaluation using T2T-HG002

The overarching goal of TTT is to provide plausible sequence models for residual tangles that conventional graph traversal algorithms fail to resolve. To test the ability of TTT to generate relevant traversals, we first extracted all tangles from a Verkko assembly of the HG002 sample and compared TTT with Verkko's multiplex de Bruijn graph-based repeat resolution[3] on the same tangles, as well as with the HG002[24] reference sequence.

For evaluation, we used a HiFi-only graph, which allows us to compare against Verkko's ONT-based repeat resolution on exactly the same tangles. We defined tangles in the graph based solely on the graph structure (Methods S2). We filtered out all tangles that had neither two nor four boundary nodes, as well as tangles with four boundary nodes that were not resolved or scaffolded by Verkko (for which we cannot infer boundary-node matching from Verkko's scaffolds). On the remaining 220 tangles, we ran TTT with all parameters set to their default values. Two runs crashed due to incorrectly identified tangle borders, and 218 succeeded, providing 397 traversal paths (179 tangles were traversed by two genomic paths).

We compared TTT's traversals with those inferred from the Verkko 2.2.1 results for the same tangles. In most cases (363 of 397; 91.4%), they were identical. In three cases, detected tangle borders did not belong to the same Verkko paths. There were five cases where we could not compare TTT against Verkko due to gaps in the final Verkko contigs and one case where Verkko closed the gap in the assembly graph by adding a sequence from ONT reads (an option not available to TTT). For the remaining 25 cases where Verkko and TTT traversals differed, we compared them by aligning corresponding sequences to the HG002 v.1.1

reference with minimap2[25] using minimap's alignment score for the best alignment as the measure of quality (Table S1). In 8 cases, TTT's resolution scored better than Verkko's, while in 17, it was worse. We manually verified all cases where TTT's score differed substantially from Verkko's and confirmed that all those cases were due to uneven ONT coverage, which led to errors in multiplicity estimation. For all tangles in HG002, the final alignment-based score is identical for all ten initial Eulerian paths, which is the default number of random starts. Thus, we suppose that it is highly unlikely (although possible) that these scores and final paths correspond to local but not global optima.

This test focused only on those tangles where a Verkko resolution was available and therefore is biased toward simpler, resolvable tangles (Table S2). We do not generally expect TTT to outperform a state-of-the-art T2T genome assembler that is able to use more data (coverage information from different technologies and Hi-C data). While this test does not demonstrate an improvement over Verkko's resolution, the fact that for most tangles, TTT's results match Verkko's confirms that TTT is consistent and comparable with existing resolution methods and serves as a sanity check.

Performance on the T2T-giraffe dataset

To demonstrate TTT's applicability to more diverse datasets, we evaluated its performance on a giraffe HiFi + ONT duplex + ONT UL + Hi-C assembly.[21] To focus on relatively complex tangles while retaining a reliable reference for comparison, we designed the experiment as follows.

We used a Verkko v.2.2.1 assembly (hereafter "verkko-normal") generated with the full dataset as a baseline. HiFi and ONT duplex were used together to construct the initial assembly graph. In addition, we constructed a verkko-gapped assembly using the same HiFi, ONT duplex, and Hi-C reads but excluding all ONT ultra-long reads longer than 100 kb. Due to the absence of ultra-long ONT reads and the presence of Hi-C data, Verkko was forced to introduce more jumps across tangled regions. The resulting gapped assembly contained 51 scaffold gaps. Using these gaps and the corresponding assembly graph, we identified 12 two-haplotype and 14 one-haplotype tangles (Table S3) and resolved them using TTT. As in the T2T-HG002 analysis, we used a HiFi-only graph, enabling comparison with Verkko's ONT-based repeat resolution.

Of the 38 gaps resolved by TTT, only 19 (5 in one-haplotype tangles and 14 in two-haplotypes) were also fully resolved in the verkko-normal assembly. Among these, 16 were resolved identically to the paths in the assembly graph, while 3 (two single-haplotype gaps and one haplotype from a two-haplotype tangle) differed from Verkko's traversal. These results indicate that TTT produces traversals consistent with a state-of-the-art genome assembler when both approaches yield solutions and provide some confidence in TTT's solutions in cases lacking assembler-derived ground truth, although their accuracy cannot be directly assessed.

Tangle resolution in the zebra finch near the T2T genome

Given the success of our reference-based comparison, we set out to test TTT by resolving the remaining gaps in a recently published near-T2T zebra finch (*Taeniopygia guttata*) genome.[26] The current reference (RefSeq: GCA_048771995.1) is based on a Verkko 2.2.1[6] assembly. The assembler's output contained 4 gaps on the chr Z scaffold associated with highly collapsed repetitive sequences flanking the gaps (Figures 2A, 2B, S1A, and S1B). Two of these tangles were resolved using standard approaches, that is, by manual graph curation and by patching using a different Verkko assembly that was able to span the gap.

Two residual tangles were considered too complex for manual curation by the zebra finch T2T project, motivating TTT development (Figure 2B). Of note, 3 of the 4 gaps, including the two tangles not resolvable by manual curation, were also not resolved when using the popular assembler hifiasm v.0.23.0,[4] with the same HiFi and ONT data as input (Figure S2), with multiple short contigs aligning to TTT's models. TTT was run on these two unresolved tangles in Verkko's assembly. The first tangle contained 45 edges, and the second tangle contained 32 edges. In both cases, the tangles were modeled with TTT, and then the sequence was incorporated in the existing reference (2.6 and 1.8 Mbp for the two tangles, respectively). Validation using NucFlag of the original assembly and the final gap-filled result showed a dramatic reduction in read pileups and secondary allele frequency (Figures 2C, 2D, S1C, and S1D). DEGAP filled the same two gaps, but the sequence was shorter than TTT (0.4 and 0.1 Mbp), and NucFlag flagged the resolved regions as collapsed (Figure S3).

The TTT reconstruction enables the study of this region of the zebra finch chrZ (Figure 3). Gene annotation with miniprot of the whole chromosome shows multiple clusters of ampliconic gene arrays dominated by p21-activated serine/threonine kinase 3-like (*PAK3L*) gene sequences (Figure 3A). Strikingly, all 4 original gaps coincide with *PAK3L* ampliconic gene arrays. Focusing on the regions resolved by TTT, the first tangle contains 12 units of median length 268 kbp, and the second contains 35 units of median length 59 kbp, well beyond the resolution potential of bridging-read-based gap-closing tools (Figures 3B and 3C). Miniprot annotations of *PAK3L* as queries reveal exactly 200 copies of the *PAK3L* gene organized in 10 main clusters (Figure 3A). Of the 200 copies, only 4 show frameshift issues, suggesting that they may largely be functional.

The *PAK3L* ampliconic gene arrays are of particular interest for the zebra finch biology, as 31 *PAK3L* genes were previously annotated in the zebra finch genome, with 11 copies associated with chrZ,[29] of which 5 were assigned to coordinates, while 6 were found on unplaced sequences.[28] Another 17 copies could not be assigned to any chromosome. Reported sequence similarity was high, and the duplications were all considered to have occurred within the past 5 mya. When the gene predictions are assigned to the best *PAK3L* gene given their sequence homology, they reveal a pattern in which specific *PAK3L* genes are associated with different clusters (Figure 3D). For instance, the first cluster is using only *PAK3L-6*, *PAK3L-11*, and *PAK3L-17*. A clear tendency for more similar gene copies to colocalize is also visible throughout the chromosome. Importantly, the two initially unresolved tangles that were modeled by TTT share the same *PAK3L-9* gene in multiple copies, with the second tangle having the highest number of the same *PAK3L* genes in the whole chromosome. This may explain why these regions

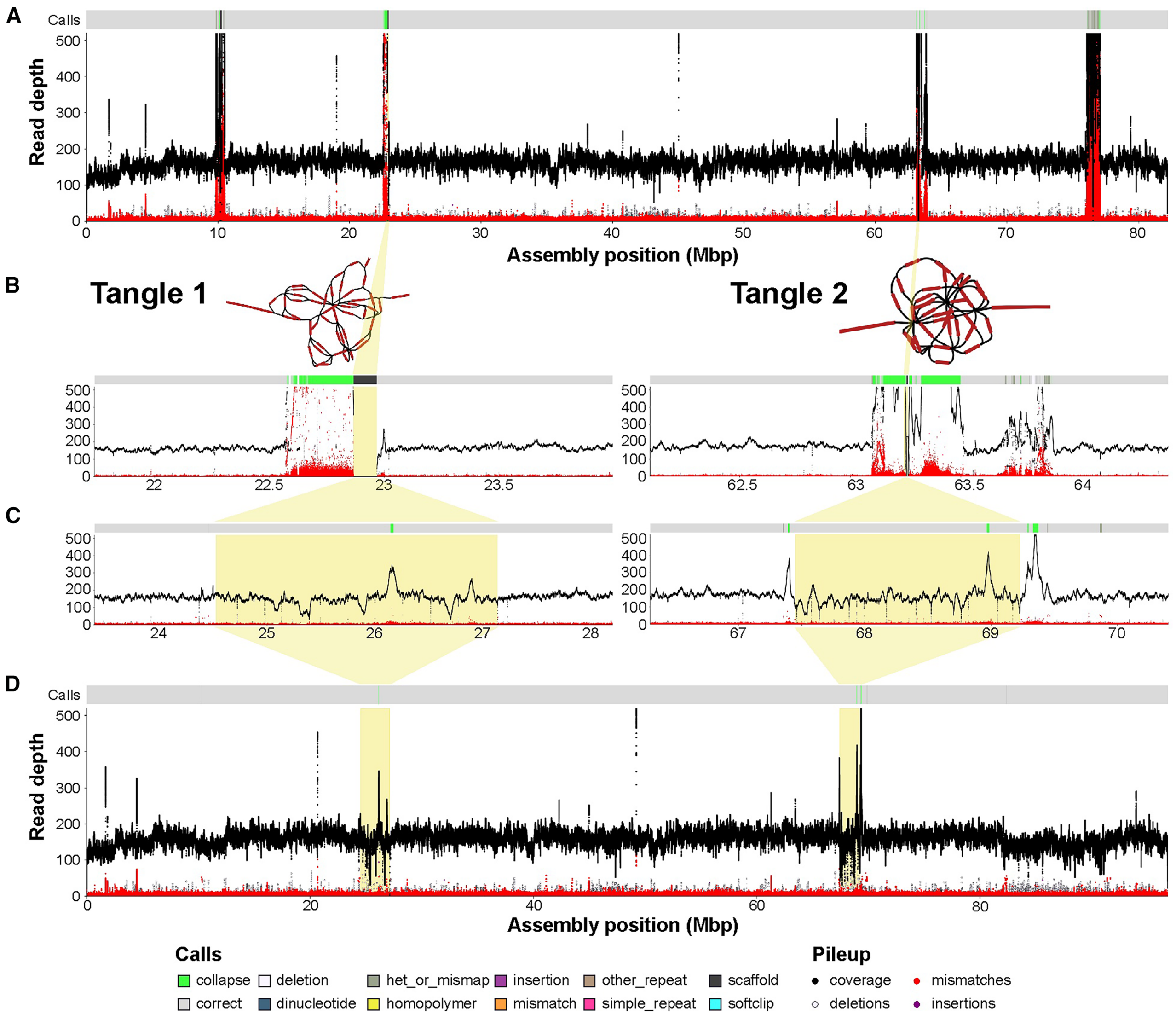

Figure 2. Coverage plots for two zebra finch chromosome Z tangles before and after repeat resolution

(A) NucFlag ONT coverage plots for zebra finch chromosome Z before resolving the gaps (GenBank: NC_133063.1), two tangle regions (B) before and (C) after running TTT, and (D) zebra finch chromosome Z after resolving the four gaps. The start and end positions for the plotting region in (B) and (C) are the same for each tangle, with the tangles highlighted in yellow. The primary read coverage is shown in black, while the secondary allele frequency is shown in red. Peaks or drops in coverage (black line) indicate assembly issues, either collapse (peaks) or mis-joins (drops). However, some variation is expected due to coverage biases in sequencing data. Peaks in the secondary allele (red) support classifying a collapse, as they indicate that a large subset of reads disagree with the assembly, most likely because these reads belong to a different repeat copy. Figure S1 shows results using PacBio HiFi sequencing data. Figure S2 shows hifiasm alignments against the TTT resolution. Figure S3 shows PacBio HiFi and ONT coverage of DEGAP resolutions. The bar above each coverage plot indicates automatically annotated errors in the assembly, colored by error type. The highlighted regions show a dramatic decrease after TTT resolution (C).

were particularly recalcitrant to assembly, while the regions with the two clusters with the largest numbers of copies overall were more diverse and did not require sequence modeling with TTT.

PAK3L genes in different clusters also appear to have evolved a different intronic content, and other cluster-specific gene duplications are sometimes present (Figure 3B), which may explain why they appeared as completely distinct in the assembly graph. These other duplications include YTHDC2-like, heat shock factor protein 5-like, and coiled-coiled 171-like genes in cluster 3, as well as inositol triphosphate receptor-interacting protein-like genes and potassium channel subfamily K member 16-like genes in cluster 8. Interestingly, in contrast to previous reports[30] that described songbird *YTHDC2* as highly truncated and *YTHDC2L1* as retaining only the HA2 and OB-fold domains, the genes identified here contained all the functional domains (DEAD, HA2, Helicase_C, OB-fold, R3H, and YTH). Relative to *PAK3*, which resides on chr4A, *PAK3L* paralogs were shown to lack the 5′ exons encoding the N-terminal regulatory domain.[28] However, at least seven *PAK3L* paralogs showed evidence of expression, primarily in the testis but also in the brain, even

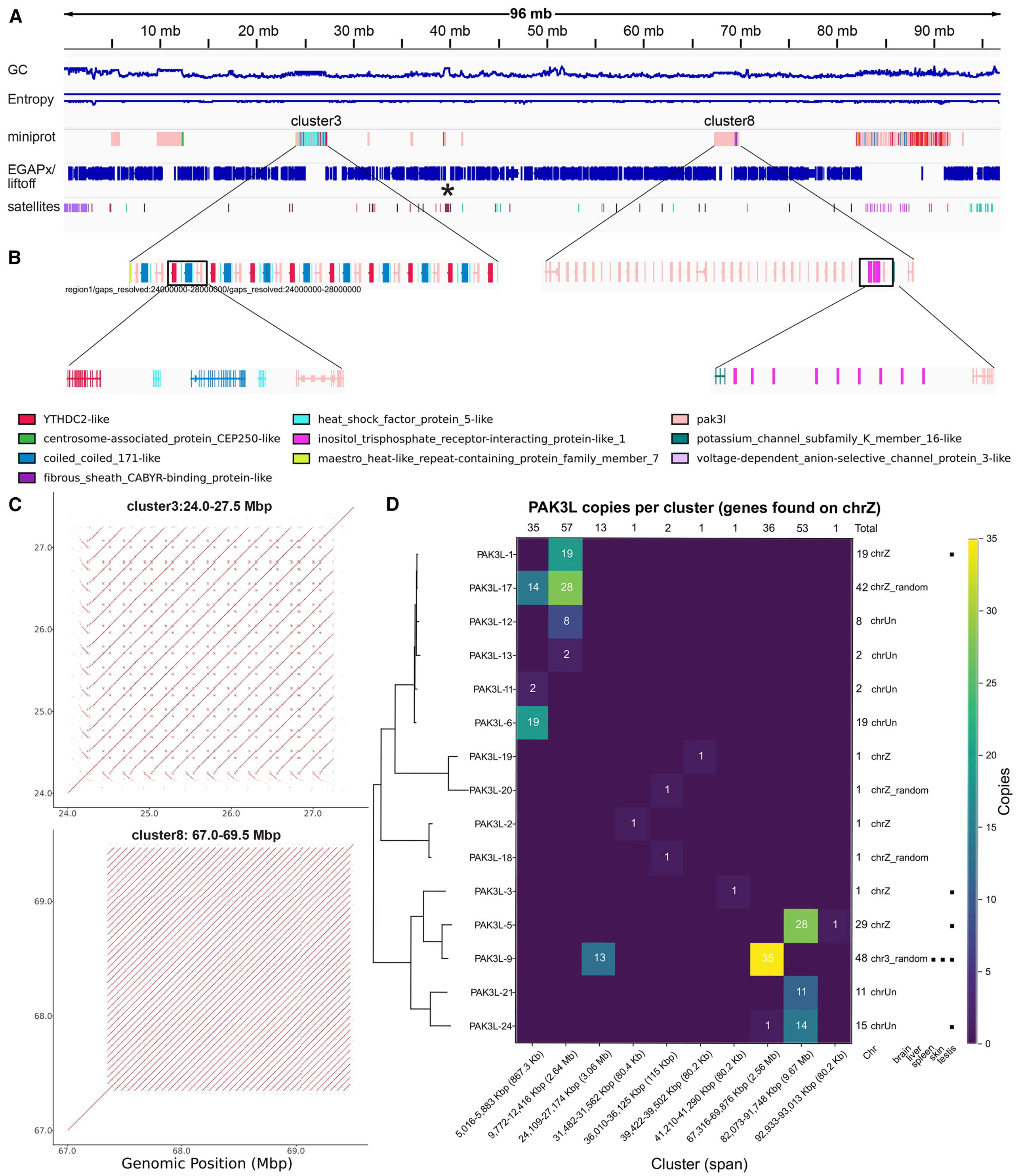

Figure 3. *PAK3L* clusters on the zebra finch chromosome Z

(A) Integrative Genomics Viewer (IGV)[27] representation of chromosome Z (chrZ) after gap resolution. Tracks include GC content, sequence entropy, miniprot alignments using proteins identified by EGAPx gene annotations as query, Liftoff-transferred EGAPx gene annotations from previous T2T assembly for chrZ, and tandem repeat annotations. The centromere is indicated by an asterisk. Ten different clusters of the *PAK3L* genes are visible in the miniprot track.

(legend continued on next page)

though *PAK3* expression is considered restricted to the central nervous system (Figure 3D). In addition, nine *PAK3L* paralogs were previously reported to be under positive selection.[28] Interestingly, *PAK3L-9*, i.e., the copy present in the two tangles resolved by TTT, was reported to be expressed in multiple tissues (testis, spleen, and skin). While the function of *PAK3L* remains to be elucidated, it is likely to contribute to songbird-specific biology. The resolution of the zebra finch chrZ clearly shows how TTT enabled the study of this biology at chromosome resolution.

DISCUSSION

Here, we describe a method for constructing model sequences for the most complex genomic regions that are left unresolved even with the state-of-the-art T2T assemblers and sequencing technologies. TTT searches for the complete traversal that best fits the input data rather than the partial but more conservative outputs of most genome assemblers. Separating the edge multiplicity determination problem and the search for the best traversal itself allows us to find an approximate solution for both using common optimization techniques—mixed integer linear programming and gradient descent optimization.

We have shown that our evaluation metrics allow us to assign an *a priori* score or confidence measure consistent with reference-based evaluation. However, it remains unclear whether these metrics should be interpreted as an evaluation of the tangle-traversing sequence itself or merely of the intrinsic difficulty of the problem. The coverage-based metric clearly depends on coverage uniformity, whereas the read-based metric depends on tangle complexity and the number of graph edges involved. Consequently, we cannot provide any recommendation for metric values that could be used as thresholds separating plausible from non-plausible traversals.

The described algorithms can be extended to incorporate multiple sequencing technologies. One clear way to improve TTT's traversal is to use coverage information from different platforms simultaneously when determining multiplicities or to modify the scoring function to account for Hi-C or trio-based information. Another clear place for improvement is the support for constructing model sequences for multi-chromosomal repeats, which can be done in a similar way to the two-haplotype-repeat resolution (Methods S1).

TTT relies on coverage information for copy-number estimation. However, certain repeat arrays are known to exhibit substantial sequencing coverage bias,[1] and copy number may therefore be misestimated in some cases. Nevertheless, TTT is designed to include all edges with sufficient coverage in the final traversal, unless doing so would contradict the assembly graph or coverage constraints. As a result, repeat variants are preserved, although their ordering may be incorrect. We consider this preferable to representing such regions as gaps (missing data) or as fragmented contigs, which are difficult to analyze using standard sequence analysis tools. As an example of this utility, by closing the remaining gaps in zebra finch chrZ using TTT, we were able to precisely map multiple ampliconic gene arrays, assigning distinct copies of the duplicated genes to their genomic loci on chrZ while also quantifying them. Zebra finch chrZ harbors large chromosomal inversions[31] that have been shown to be strikingly associated with phenotypic variation in siring success.[32–34] It did not escape our attention that such inversions appear to colocalize with the ampliconic gene arrays and that the genes involved tend to be expressed in the testis.[28,35] Further research will be needed to precisely characterize these inversions at the sequence level and determine their potential phenotypic impact.

In our benchmark, a total of 179 of 218 (82%) T2T-HG002 tangles were diploid, and TTT's performance on 163 (91%) of these diploid tangles is equivalent to a modern diploid genome assembler (Verkko), yielding exactly the same local phasing consistency. However, for more complex diploid tangles that were unresolved by a T2T assembler, a common scenario involves sequences present at different copy numbers between haplotypes, for example, due to a duplication event in only one haplotype. Failure of the assembler to resolve such regions typically indicates ambiguity in assigning repeat copies to haplotypes or in estimating haplotype-specific copy numbers. Accordingly, while TTT produces traversals consistent with read alignments—and, in ambiguous cases, ensures that each haplotype contains at least one copy of a repeat—caution should be exercised when using these traversals to infer haplotype-specific copy number or exact per-haplotype duplication status.

We have described an algorithmic approach that is expected to close some of the remaining gaps in T2T scaffolds. Currently, TTT cannot be used to model inter-chromosomal repeats (e.g., rDNA tangles in human genomes), and it will not help in cases where gaps are caused by reduced coverage rather than complex repetitive structure. Although not applicable to all gaps in a genome assembly, we believe it represents an important step toward truly T2T genomes and the study of complex regions of the genome that are otherwise not represented in the assemblies and therefore not accessible to investigation.

Limitations of the study

TTT is not universal and cannot be applied to every gap in an assembly. Detailed requirements are listed in the STAR Methods section. Across 8,532 gaps in 227 HPRC human assemblies generated with Verkko v.2.3, 48.9% gaps were TTT applicable (Table S4). The heuristic nature of the TTT algorithm results in traversals that should be considered model sequences of *a priori* lower quality than standard assembler output. This distinction can be represented in the final assembly in different ways—through quality scores, annotation, or simply by separating the model sequence from the assembled scaffold in a FASTA file.

(B) Overview of the cluster 3 and 8 regions (gaps filled by TTT), with an inset highlighting the duplicated gene structure. Cluster 8 shows duplication of the *PAK3L* gene and other genes, such as the inositol trisphosphate receptor-interacting protein-like 1 gene.

(C) Self-similarity dot plots of the two clusters.

(D) Heatmap showing the copy number of each *PAK3L* gene across clusters, together with a schematic summary of the genes for previously reported chromosomal localization and tissue-specific expression.[28]

We emphasize that such a distinction should be represented *somehow*; therefore, we intentionally do not provide automatically "patched" T2T scaffolds as output so that users must make a conscious decision about how to incorporate the traversals.

RESOURCE AVAILABILITY

Lead contact

Requests for further information and resources should be directed to and will be fulfilled by the lead contact, Sergey Koren (sergekoren@gmail.com).

Materials availability

No new data were generated for this study.

Data and code availability

- TTT is available on GitHub (https://github.com/marbl/TTT). Version tagged v.0.1.1 (https://doi.org/10.5281/zenodo.20836977) was used for benchmarking in this study.
- Sequencing data for the HG002 dataset are available at HPRC's Amazon cloud at https://s3-us-west-2.amazonaws.com/human-pangenomics/index.html. Giraffe sequencing data are available under BioProject: PRJNA1367195.
- Raw sequencing data for zebra finch are available in the NCBI SRA under BioProject: PRJNA1241433. The fully resolved sequence for chrZ is available at https://genomeark.s3.amazonaws.com/index.html?prefix=species/Taeniopygia_guttata/bTaeGut7/assembly_verkko_0.1/patching/.

ACKNOWLEDGMENTS

This research was supported by the Intramural Research Program of the National Human Genome Research Institute of the National Institutes of Health (NIH) and by the National Human Genome Research Institute of the National Institutes of Health under award number R03HG013362. The contributions of the NIH authors are considered works of the United States government. The findings and conclusions presented in this paper are those of the authors and do not necessarily reflect the views of the NIH or the US Department of Health and Human Services. This work utilized the computational resources of the NIH HPC Biowulf cluster (https://hpc.nih.gov). The authors thank Arang Rhie, Nancy Hansen, Juhyun Kim, and Pavel Pevzner for helpful discussions; Ksenia Krasheninnikova, Ben Rosen, Sarah E. Fumagalli, Brendan Pinto, Jiadong Lin, and Jeffrey Schoenebeck for testing early versions of TTT and providing valuable feedback on its performance; and Keisuke K. Oshima for his help on NucFlag analyses.

AUTHOR CONTRIBUTIONS

D.A. developed the TTT algorithm and wrote the initial manuscript draft. G.F., Y.C., and M.S. performed the analysis of zebra finch repeat arrays. D.A., Y.C., M.S., and G.F. generated the figures. A.M.P. and G.F. provided project supervision and coordination. G.F. formulated the tangle traversing problem and supervised analysis of zebra finch repeat arrays. S.K. supervised the development of the TTT algorithm and implementation. All authors edited the manuscript. All authors read and approved the final manuscript.

DECLARATION OF INTERESTS

The authors declare no competing interests.

STAR★METHODS

Detailed methods are provided in the online version of this paper and include the following:

- KEY RESOURCES TABLE
- METHOD DETAILS
  - TTT algorithm
  - Identifying multiplicities of edges in the traversing path
  - Finding the optimal path with given edge multiplicities
  - Requirements
  - Gap resolution in zebra finch chr Z
  - Gene models identification
  - DEGAP analysis

SUPPLEMENTAL INFORMATION

Supplemental information can be found online at https://doi.org/10.1016/j.xgen.2026.101323.

Received: March 6, 2026
Revised: July 8, 2026
Accepted: July 9, 2026
Published: August 6, 2026

REFERENCES

1. Nurk, S., Koren, S., Rhie, A., Rautiainen, M., Bzikadze, A.V., Mikheenko, A., Vollger, M.R., Altemose, N., Uralsky, L., Gershman, A., et al. (2022). The complete sequence of a human genome. Science *376*, 44–53.
2. Cheng, H., Concepcion, G.T., Feng, X., Zhang, H., and Li, H. (2021). Haplotype-resolved de novo assembly using phased assembly graphs with hifiasm. Nat. Methods *18*, 170–175. https://doi.org/10.1038/s41592-020-01056-5.
3. Rautiainen, M., Nurk, S., Walenz, B.P., Logsdon, G.A., Porubsky, D., Rhie, A., Eichler, E.E., Phillippy, A.M., and Koren, S. (2023). Telomere-to-telomere assembly of diploid chromosomes with Verkko. Nat. Biotechnol. *41*, 1474–1482. https://doi.org/10.1038/s41587-023-01662-6.
4. Cheng, H., Asri, M., Lucas, J., Koren, S., and Li, H. (2024). Scalable telomere-to-telomere assembly for diploid and polyploid genomes with double graph. Nat. Methods *21*, 967–970. https://doi.org/10.1038/s41592-024-02269-8.
5. Lorig-Roach, R., Meredith, M., Monlong, J., Jain, M., Olsen, H.E., McNulty, B., Porubsky, D., Montague, T.G., Lucas, J.K., Condon, C., et al. (2024). Phased nanopore assembly with Shasta and modular graph phasing with GFAse. Genome Res. *34*, 454–468. https://doi.org/10.1101/gr.278268.123.
6. Antipov, D., Rautiainen, M., Nurk, S., Walenz, B.P., Solar, S.J., Phillippy, A.M., and Koren, S. (2025). Verkko2 integrates proximity-ligation data with long-read De Bruijn graphs for efficient telomere-to-telomere genome assembly, phasing, and scaffolding. Genome Res. *35*, 1583–1594. https://doi.org/10.1101/gr.280383.124.
7. Li, H., and Durbin, R. (2024). Genome assembly in the telomere-to-telomere era. Nat. Rev. Genet. *25*, 658–670. https://doi.org/10.1038/s41576-024-00718-w.
8. Rautiainen, M. (2024). Ribotin: automated assembly and phasing of rDNA morphs. Bioinformatics *40*, btae124. https://doi.org/10.1093/bioinformatics/btae124.
9. Koren, S., Rhie, A., Walenz, B.P., Dilthey, A.T., Bickhart, D.M., Kingan, S.B., Hiendleder, S., Williams, J.L., Smith, T.P.L., and Phillippy, A.M. (2018). De novo assembly of haplotype-resolved genomes with trio binning. Nat. Biotechnol. *36*, 1174–1182.
10. Garg, S., Fungtammasan, A., Carroll, A., Chou, M., Schmitt, A., Zhou, X., Mac, S., Peluso, P., Hatas, E., Ghurye, J., et al. (2021). Chromosome-scale, haplotype-resolved assembly of human genomes. Nat. Biotechnol. *39*, 309–312. https://doi.org/10.1038/s41587-020-0711-0.
11. Yoo, D., Rhie, A., Hebbar, P., Antonacci, F., Logsdon, G.A., Solar, S.J., Antipov, D., Pickett, B.D., Safonova, Y., Montinaro, F., et al. (2025). Complete sequencing of ape genomes. Nature *641*, 401–418. https://doi.org/10.1038/s41586-025-08816-3.
12. Olagunju, T.A., Pospelova, M., Schwartz, J.C., Mousel, M.R., Piel, L.M.W., Grossman, P.C., Huyvaert, K.P., Kuhn, K.L., Raihan, T., Stegemiller, M.R.,

et al. (2025). Bighorn sheep T2T genome assembly reveals differences in immune genes: a potential cause of high morbidity due to respiratory pathogens. Preprint at bioRxiv. https://doi.org/10.1101/2025.09.30.679298.

13. Garg, V., Bohra, A., Mascher, M., Spannagl, M., Xu, X., Bevan, M.W., Bennetzen, J.L., and Varshney, R.K. (2024). Unlocking plant genetics with telomere-to-telomere genome assemblies. Nat. Genet. *56*, 1788–1799. https://doi.org/10.1038/s41588-024-01830-7.
14. Miga, K.H., and Eichler, E.E. (2023). Envisioning a new era: Complete genetic information from routine, telomere-to-telomere genomes. Am. J. Hum. Genet. *110*, 1832–1840. https://doi.org/10.1016/j.ajhg.2023.09.011.
15. Xu, G.-C., Xu, T.-J., Zhu, R., Zhang, Y., Li, S.-Q., Wang, H.-W., and Li, J.-T. (2019). LR_Gapcloser: a tiling path-based gap closer that uses long reads to complete genome assembly. GigaScience *8*, giy157. https://doi.org/10.1093/gigascience/giy157.
16. Xu, M., Guo, L., Gu, S., Wang, O., Zhang, R., Peters, B.A., Fan, G., Liu, X., Xu, X., Deng, L., and Zhang, Y. (2020). TGS-GapCloser: A fast and accurate gap closer for large genomes with low coverage of error-prone long reads. GigaScience *9*, giaa094. https://doi.org/10.1093/gigascience/giaa094.
17. Zimin, A.V., and Salzberg, S.L. (2022). The SAMBA tool uses long reads to improve the contiguity of genome assemblies. PLoS Comput. Biol. *18*, e1009860. https://doi.org/10.1371/journal.pcbi.1009860.
18. Huang, Y., Wang, Z., Schmidt, M.A., Su, H., Xiong, L., and Zhang, J. (2024). DEGAP: Dynamic elongation of a genome assembly path. Brief. Bioinform. *25*, bbae194. https://doi.org/10.1093/bib/bbae194.
19. Zhao, X., Liu, F., and Pan, W. (2024). Comprehensive evaluation of genome gap-filling tools utilizing long reads. Genes *15*, 127. https://doi.org/10.3390/genes15010127.
20. Chu, Y., Huang, Z., Shao, C., Guo, S., Yang, Y., Yu, X., Wang, J., Tian, Y., Chen, J., Li, R., et al. (2025). Approaching an Error-Free Diploid Human Genome Using a Support-Based Validation Framework. Preprint at bioRxiv. https://doi.org/10.1101/2025.08.01.667781.
21. Kim, J., Rosen, B.D., Fumagalli, S.E., Kuhn, K.L., Long, A., Schoenebeck, J.J., Schwartz, H., Wu-Cavener, L., Zimin, A.V., Cavener, D.R., et al. (2026). Finishing a complete giraffe genome from telomere to telomere with Verkko-Fillet. Cell Genomics *6*, 101279. https://doi.org/10.1016/j.xgen.2026.101279.
22. Meyer, R.R. (1974). On the existence of optimal solutions to integer and mixed-integer programming problems. Math. Program. *7*, 223–235. https://doi.org/10.1007/bf01585518.
23. Meyer, R.R. (1975). Integer and mixed-integer programming models: General properties. J. Optim. Theory Appl. *16*, 191–206. https://doi.org/10.1007/bf01262932.
24. Hansen, N.F., Dwarshuis, N., Ji, H.J., Rhie, A., Loucks, H., Logsdon, G.A., Vollger, M.R., Storer, J.M., Kim, J., Adam, E., et al. (2026). A complete diploid human genome benchmark for personalized genomics. Cell *189*, 1–19. https://doi.org/10.1016/j.cell.2026.06.016.
25. Li, H. (2018). Minimap2: pairwise alignment for nucleotide sequences. Bioinformatics *34*, 3094–3100. https://doi.org/10.1093/bioinformatics/bty191.
26. Formenti, G., Jain, N., Medico, J.A., Sollitto, M., Antipov, D., Barcellos, S., Biegler, M., Borges, I., Chang, J.K., Chen, Y., et al. (2026). The complete genome of a songbird. Cell *189*, 1–24. https://doi.org/10.1016/j.cell.2026.07.018.
27. Robinson, J.T., Thorvaldsdóttir, H., Winckler, W., Guttman, M., Lander, E.S., Getz, G., and Mesirov, J.P. (2011). Integrative genomics viewer. Nat. Biotechnol. *29*, 24–26. https://doi.org/10.1038/nbt.1754.
28. Kong, L., Lovell, P.V., Heger, A., Mello, C.V., and Ponting, C.P. (2010). Accelerated evolution of PAK3- and PIM1-like kinase gene families in the zebra finch, Taeniopygia guttata. Mol. Biol. Evol. *27*, 1923–1934. https://doi.org/10.1093/molbev/msq080.
29. Warren, W.C., Clayton, D.F., Ellegren, H., Arnold, A.P., Hillier, L.W., Künstner, A., Searle, S., White, S., Vilella, A.J., Fairley, S., et al. (2010). The genome of a songbird. Nature *464*, 757–762. https://doi.org/10.1038/nature08819.
30. Wirthlin, M., Lovell, P.V., Jarvis, E.D., and Mello, C.V. (2014). Comparative genomics reveals molecular features unique to the songbird lineage. BMC Genom. *15*, 1082. https://doi.org/10.1186/1471-2164-15-1082.
31. Itoh, Y., Kampf, K., Balakrishnan, C.N., and Arnold, A.P. (2011). Karyotypic polymorphism of the zebra finch Z chromosome. Chromosoma *120*, 255–264. https://doi.org/10.1007/s00412-010-0308-3.
32. Knief, U., Hemmrich-Stanisak, G., Wittig, M., Franke, A., Griffith, S.C., Kempenaers, B., and Forstmeier, W. (2016). Fitness consequences of polymorphic inversions in the zebra finch genome. Genome Biol. *17*, 199. https://doi.org/10.1186/s13059-016-1056-3.
33. Kim, K.-W., Bennison, C., Hemmings, N., Brookes, L., Hurley, L.L., Griffith, S.C., Burke, T., Birkhead, T.R., and Slate, J. (2017). A sex-linked supergene controls sperm morphology and swimming speed in a songbird. Nat. Ecol. Evol. *1*, 1168–1176. https://doi.org/10.1038/s41559-017-0235-2.
34. Knief, U., Forstmeier, W., Pei, Y., Ihle, M., Wang, D., Martin, K., Opatová, P., Albrechtová, J., Wittig, M., Franke, A., et al. (2017). A sex-chromosome inversion causes strong overdominance for sperm traits that affect siring success. Nat. Ecol. Evol. *1*, 1177–1184. https://doi.org/10.1038/s41559-017-0236-1.
35. Viitaniemi, H.M., Leder, E.H., Kauzál, O., Stopková, R., Stopka, P., Lifjeld, J.T., and Albrecht, T. (2024). Impact of Z chromosome inversions on gene expression in testis and liver tissues in the zebra finch. Mol. Ecol. *33*, e17236. https://doi.org/10.1111/mec.17236.
36. Shumate, A., and Salzberg, S.L. (2021). Liftoff: accurate mapping of gene annotations. Bioinformatics *37*, 1639–1643. https://doi.org/10.1093/bioinformatics/btaa1016.
37. Gao, S., Oshima, K.K., Chuang, S.-C., Loftus, M., Montanari, A., Gordon, D.S., Hsieh, P., Konkel, M.K., PingHsun, H., Miriam, K.K., et al. (2025). A global view of human centromere variation and evolution. Preprint at bioRxiv. https://doi.org/10.64898/2025.12.09.693231.
38. Danecek, P., Bonfield, J.K., Liddle, J., Marshall, J., Ohan, V., Pollard, M.O., Whitwham, A., Keane, T., McCarthy, S.A., Davies, R.M., and Li, H. (2021). Twelve years of SAMtools and BCFtools. GigaScience *10*, giab008. https://doi.org/10.1093/gigascience/giab008.
39. Kiełbasa, S.M., Wan, R., Sato, K., Horton, P., and Frith, M.C. (2011). Adaptive seeds tame genomic sequence comparison. Genome Res. *21*, 487–493. https://doi.org/10.1101/gr.113985.110.
40. Li, H. (2023). Protein-to-genome alignment with miniprot. Bioinformatics *39*, btad014. https://doi.org/10.1093/bioinformatics/btad014.
41. Sweeten, A.P., Schatz, M.C., and Phillippy, A.M. (2024). ModDotPlot-rapid and interactive visualization of tandem repeats. Bioinformatics *40*, btae493. https://doi.org/10.1093/bioinformatics/btae493.
42. Huerta-Cepas, J., Forslund, K., Coelho, L.P., Szklarczyk, D., Jensen, L.J., von Mering, C., and Bork, P. (2017). Fast genome-wide functional annotation through orthology assignment by eggNOG-mapper. Mol. Biol. Evol. *34*, 2115–2122. https://doi.org/10.1093/molbev/msx148.
43. Chikhi, R., Limasset, A., Jackman, S., Simpson, J.T., and Medvedev, P. (2015). On the representation of de Bruijn graphs. J. Comput. Biol. *22*, 336–352. https://doi.org/10.1089/cmb.2014.0160.
44. Li, H. (2016). Minimap and miniasm: fast mapping and de novo assembly for noisy long sequences. Bioinformatics *32*, 2103–2110. https://doi.org/10.1093/bioinformatics/btw152.
45. Bankevich, A., Bzikadze, A.V., Kolmogorov, M., Antipov, D., and Pevzner, P.A. (2022). Multiplex de Bruijn graphs enable genome assembly from long, high-fidelity reads. Nat. Biotechnol. *40*, 1075–1081. https://doi.org/10.1038/s41587-022-01220-6.
46. Chang, X., Eizenga, J., Novak, A.M., Sirén, J., and Paten, B. (2020). Distance indexing and seed clustering in sequence graphs. Bioinformatics *36*, i146–i153. https://doi.org/10.1093/bioinformatics/btaa446.

47. Rieber, N., Zapatka, M., Lasitschka, B., Jones, D., Northcott, P., Hutter, B., Jäger, N., Kool, M., Taylor, M., Lichter, P., et al. (2013). Coverage bias and sensitivity of variant calling for four whole-genome sequencing technologies. PLoS One *8*, e66621. https://doi.org/10.1371/journal.pone.0066621.

48. Hon, T., Mars, K., Young, G., Tsai, Y.-C., Karalius, J.W., Landolin, J.M., Maurer, N., Kudrna, D., Hardigan, M.A., Steiner, C.C., et al. (2020). Highly accurate long-read HiFi sequencing data for five complex genomes. Preprint at bioRxiv. https://doi.org/10.1101/2020.05.04.077180.

49. Delahaye, C., and Nicolas, J. (2021). Sequencing DNA with nanopores: Troubles and biases. PLoS One *16*, e0257521. https://doi.org/10.1371/journal.pone.0257521.

50. Carvalho, A.B., Kim, B.Y., and Uno, F. (2026). Strong bias in long-read sequencing prevents assembly of Drosophila melanogaster Y-linked genes. Genome Res. *36*, 71–82. https://doi.org/10.1101/gr.280604.125.

51. Papadimitriou, C.H. (1981). On the complexity of integer programming. J. ACM *28*, 765–768. https://doi.org/10.1145/322276.322287.

52. Hierholzer, C., and Wiener, C. (1873). Über die Möglichkeit, einen Linienzug ohne Wiederholung und ohne Unterbrechung zu umfahren. Math. Ann. *6*, 30–32.

53. Rautiainen, M., and Marschall, T. (2020). GraphAligner: rapid and versatile sequence-to-graph alignment. Genome Biol. *21*, 253. https://doi.org/10.1186/s13059-020-02157-2.

54. Aho, A.V., and Corasick, M.J. (1975). Efficient string matching. Commun. ACM *18*, 333–340. https://doi.org/10.1145/360825.360855.

55. Vollger, M.R., Dishuck, P.C., Sorensen, M., Welch, A.E., Dang, V., Dougherty, M.L., Graves-Lindsay, T.A., Wilson, R.K., Chaisson, M.J.P., and Eichler, E.E. (2019). Long-read sequence and assembly of segmental duplications. Nat. Methods *16*, 88–94. https://doi.org/10.1038/s41592-018-0236-3.

STAR★METHODS

KEY RESOURCES TABLE

REAGENT or RESOURCE	SOURCE	IDENTIFIER
Software and algorithms		
Verkko v2.2.1 and v2.3	Rautiainen et al.[3]	https://github.com/marbl/verkko
DEGAP v2.0	Huang et al.[18]	https://github.com/Jianwei-Zhang/DEGAP
Minimap2 v2.30-r1287	Li[25]	https://github.com/lh3/minimap2
Liftoff v1.6.3	Shumate et al.[36]	https://github.com/agshumate/Liftoff
NucFlag v1.0.0a2	Gao et al.[37]	https://github.com/logsdon-lab/NucFlag
SAMtools v1.22	Danecek et al.[38]	https://github.com/samtools/samtools
Lastal v1648	Kiełbasa et al.[39]	https://gitlab.com/mcfrith/last
miniprot v0.18	Li[40]	https://github.com/lh3/miniprot
ModDotPlot v0.9.9	Sweeten et al.[41]	https://github.com/marbl/ModDotPlot
IGV v2.19.7	Robinson et al.[27]	https://igv.org/
eggNOG-mapper v.5.0.2	Huerta-Cepas et al.[42]	https://github.com/eggnogdb/eggnog-mapper
TTT v0.1.1	This paper	https://github.com/marbl/TTT

METHOD DETAILS

TTT algorithm

TTT considers tangle traversal as two independent sub-problems: (i) determining the multiplicities of tangle edges in the traversing path, and (ii) finding a path that is consistent with the largest number of read-to-graph alignments while respecting the edge multiplicities obtained in the first step. Both of those problems are solved heuristically. In the following we use the assembly graph representation with sequence stored on *edges* of the directed assembly graph.[43] Every scaffold can be represented by a sequence of paths in the assembly graph separated by gaps. Although the most commonly used assembly graph representation (gfa,[44]) assumes that sequence is stored in nodes, a multiplex DBG[45] in gfa format can be easily transformed into a graph with sequence on edges by considering junctions as vertices and nodes as edges.

To formally define a tangle we use an approach similar to the definition of a snarl in Chang et al.[46] We say that *n* edge pairs (x_i, y_i) define a tangle in the assembly graph if: (a) all those edges belong to the same connected component C in the assembly graph (considered as undirected graph) (b) removal of those edges splits component C into multiple connected components C_1, …C_k (c) one of those connected components C_t is significantly smaller than every other C_i (d) the end of each x_i and the start of each y_i belongs to C_t. We call C_t a *tangle*; all edges in C_t are referred to as *tangle edges,* and the pairs (x_i, y_i) are referred to as *(incoming, outgoing) boundary* edges. Figure 1 shows an example of a simple tangle and underlying genomic traversal.

TTT imposes an additional requirement for the tangles it resolves: each boundary edge x_i and y_i should be *unique* – i.e., non-repetitive and non-homozygous edges that should be present only once in a correct genome assembly. It is important to note that this graph-based definition is different from sequence-based definitions. For example a diverged copy of a repeat is typically represented by a separate edge in the assembly graph and thus is considered unique.

Since the correct assembly is unknown, in practice unique edges are identified based on their length and coverage. Informally, x_i can be considered as the last unique edge of the scaffold path preceding the gap, and y_i as the first unique edge following the gap. In contrast to snarls we must consider multiple pairs of boundary edges because a tangle edge can belong to multiple genomic paths; a common example is a repeat array shared by both haplotypes in a diploid genome.

In principle, it is possible that no unique edges occur near the gap along the scaffold path. However, this situation is uncommon in near–T2T assemblies: among 8,532 gaps across 227 assemblies, a unique pair of boundary edges could not be identified within a 5 Mbp window around the gap in fewer than 6% of cases (Table S4).

First, we describe a simplified version of the algorithm for the tangles with one incoming and one outgoing boundary edge. For simplicity, we also ignore reverse complementation. Thus, we assume that for each edge *e,* only one of *e* and *reverse_complement(e)* is reachable from the tangle boundary edges, and we do not need to distinguish multiplicities of *e* and *reverse_complement(e)*. Changes required to process two-haplotype tangles and the case of inverted repeats are described in Method S1 and Figures S4 and S5.

Identifying multiplicities of edges in the traversing path

In this step, we define optimal assignment of edge multiplicities according to the assembly graph structure and edge coverages. The primary requirement for the multiplicities is consistency with the coverage information; however, we also aim to ensure the existence of a path traversing the tangle with the assigned multiplicities, and that the resulting traversal does not discard edges with reasonable coverage.

TTT assumes that the coverage of traversals within the tangle is somehow uniform; otherwise, reliable estimation of edge multiplicities based on edge coverages would not be possible. All sequencing technologies are known to have some coverage bias,[47–50] and thus global coverage uniformity across the genome is unlikely. However, we need only local coverage uniformity, which is more likely to be observed. Moreover, with coverage information from multiple sequencing technologies available (which is a common situation for T2T assembly projects), TTT can select the one that is less biased.

We assume that each genomic path through the tangle has the same coverage, denoted later as Cov_u. The initial value for this coverage is either provided by the user or estimated as the median coverage of unique boundary edges incident to the tangle, and it can be further tuned by the optimization process.

Comparing coverage information for repetitive edges with the coverage of known unique (haploid and non-repetitive) genomic regions can provide a straightforward estimate of edge multiplicity by dividing the edge coverage by Cov_u and rounding to the nearest integer. However, due to the coverage variation, such approximations may be impossible to satisfy – for example, at a vertex with two incoming edges and one outgoing edge, all assigned multiplicity one. Therefore, in addition to encouraging coverage-based multiplicities to be as close as possible to Cov_{edge}/Cov_u, we impose a natural set of flow conservation constraints: for each vertex, the sum of multiplicities of incoming edges is expected to equal the sum of multiplicities of outgoing edges.

Lastly, to ensure completeness of the traversal, we require that all reasonably covered edges (defined as edges with coverage at least 0.5 * Cov_u) be included in the optimal traversal path at least once. Although this definition of "reasonably covered" is somewhat arbitrary, it is based on the assumption that an edge with substantial coverage is not "invented" by the assembler and therefore should not be excluded from the optimal traversal. Summarizing, with unknown integer edge multiplicities, we have a set of linear equations:

$$\sum_{e \in E_{in}(\upsilon)} \text{mult}(e) = \sum_{e \in E_{out}(\upsilon)} \text{mult}(e), \forall_\upsilon \in V$$

$$\text{mult}(e) \geq 0, \forall_e \in E$$

$$\text{mult}(e) \geq 1, \forall_e \in E_{rc}$$

$$\text{mult}(e) = 1, \forall_e \in E_b$$

With an objective to minimize the difference between the edge multiplicities and their coverage-based estimates:

$$\min \left(\sum_{e \in E} \left| \frac{\text{cov}(e)}{\text{cov}_u} - \text{mult}(e) \right| \right)$$

Where:

$\text{mult}(e)$ is the integer multiplicity of edge e.
$\text{cov}(e)$ is the coverage of edge e.
cov_u is the unique traversal coverage.
V is the set of all vertices in the tangle.
E is the set of all edges in the tangle.
$E_{in}(\upsilon)$ and $E_{out}(\upsilon)$ are the sets of incoming and outgoing edges for vertex υ.
E_{rc} is the set of reasonably covered edges.
E_b is the set of boundary edges.

This is a mixed-integer linear programming[22,23] problem formulation to determine integer multiplicities for the edges. Although MILP is known to be NP-complete,[51] there are highly optimized heuristic solvers for MILP that provide feasible locally optimal (but not guaranteed to be optimal) solutions relatively fast. In TTT we use the PuLP (https://github.com/coin-or/pulp) package and the GLPK (https://www.gnu.org/software/glpk/) solver. Note that any path through the tangle connecting the incoming and outgoing edges could yield a solution which will satisfy all constraints in (1), (2) and (4) classes. However, condition (3) may cause situations where no solution exists at all. For example, this will happen if a boundary edge (of multiplicity 1) is followed by two *reasonably covered* edges that could not both be traversed at the same time. Thus, if no solution for the original problem is found, we remove the requirement that all reasonably covered edges are used to ensure that some solution will be found. Although theoretically possible, this rollback never happened in any of the datasets used for the benchmarking.

Finding the optimal path with given edge multiplicities

First, we find just any path satisfying the multiplicities from the previous step. Lets replace each edge of multiplicity *n* with *n* multi-edges of multiplicity 1 (and the same sequence label as the original edge) and remove all edges of multiplicity 0. Thus, the resulting problem is to find the Eulerian path in this multigraph. Since we require exactly one incoming and one outgoing edge and the tangle is connected when considered as an undirected graph, such an Eulerian path exists, and can be found using Hierholzer's algorithm.[52]

There can be multiple traversal paths with the given multiplicities. The goal of this step is to provide the path that satisfies the multiplicities and is most consistent with the long-read alignments. We use read alignments provided by sequence-to-graph alignment tools such as GraphAligner,[53] and consider each read as a sequence of edge labels. We define allowed modifications of the path that preserve multiplicities, and use a gradient-descent-like approach, trying multiple modifications and accepting the modification only if it improves the score. For each path (which is also considered as a sequence of edge labels) its alignment score is defined as the number of reads which can be found as an exact substring in this path. Although inexact or partial pattern matching-based scoring can be more natural for this problem, the exact substring requirement allows the use of the fast Aho-Corasick algorithm[54] for scoring.

Let's define a *swap* operation on a path as follows: Find two non-overlapping intervals in the path that start and end on the same vertices A and B and swap the subpaths defined by those intervals (Figure S6). This operation can be considered as reordering repeat units in a repeat array.

Lemma 1: An Eulerian path in directed multigraph G can be transformed into any other Eulerian path in the same graph (with same start and end) through a sequence of swaps.

It is easy to see that one can iteratively extend the common prefix between two paths using swaps; by doing so, the length of the common prefix can be increased until the paths match entirely. Proof details can be found in Method S1.

TTT starts from a random Eulerian path, applies random swaps, and accepts a change only if it improves the score. The process terminates when no score-improving swap exists. This approach optimizes the consistency of read alignments to the chosen path while maintaining the edge multiplicities determined in step 1. Although Lemma 1 guarantees that there exists a sequence of swaps leading to a path with the optimal score, it is still possible for TTT to become trapped in a local optimum. To reduce this risk, we perform multiple iterations of this gradient-descent-like optimization using different random Eulerian paths as starting points. Moreover, there can be multiple solutions with exactly the same scores (i.e., in case of large exact repeat not traversed by any provided read alignment). Thus, even assuming the given coverage and assembly graph are accurate, the solution is not guaranteed to exactly match the genomic traversal but should be highly concordant with the underlying data.

For additional evaluation we defined two reference-free metrics for internal evaluation of TTT's results. The first is the divergence between multiplicities and coverage, normalized by coverage and number of edges, $\frac{\left(\sum_{e \in E}\left|\frac{\text{cov}(e)}{cov_u} - \text{mult}(e)\right|\right)}{|E|}$. The second is the fraction of read alignments corresponding to the tangle that are not supported by the TTT traversal (i.e., are not exact substrings in the alphabet of edge labels). On the HG002 test cases, problematic tangles tend to have higher values for both of these metrics (Figure S7).

Requirements

TTT relies on the assembly graph, read-to-graph alignments, and coverage information produced by upstream tools. Consequently, errors in the assembly graph or coverage can degrade the quality of TTT's inferred models. In particular, when a gap is caused by a coverage drop, no path may exist in the assembly graph connecting the flanking regions, precluding construction of a patch sequence.

TTT requires a defined pairing between incoming and outgoing nodes within a tangle. It is designed to patch gaps within scaffolds rather than to connect separate contigs; therefore, if such pairing is not established by the assembler (i.e., boundaries of the tangle are not scaffolded), TTT cannot be applied.

TTT also requires reliable unique incoming and outgoing edges. Although most sequence in near-T2T assemblies typically corresponds to unique edges – so that unique context is often present on both sides of a gap – this requirement can still prevent resolution in some cases, particularly for gaps located near scaffold ends (e.g., those associated with subtelomeric repeats).

In addition, the current TTT implementation does not support tangles involving more than two scaffolds (i.e., multichromosomal tangles).

We quantified the proportion of TTT-applicable gaps across 227 HPRC human assemblies generated with Verkko v2.3. Overall, 48.9% of gaps were deemed TTT-applicable. Among the remaining gaps, 27.6% lacked a connecting path in the assembly graph between scaffold ends (e.g., a sequencing coverage gap), 18.0% were associated with tangles involving more than two scaffolds, and 5.4% lacked a unique pair of flanking edges within 5 Mbp of the gap. Detailed statistics are provided in Table S4.

Gap resolution in zebra finch chr Z

Model sequences were aligned at bp resolution with lastal (http://last.cbrc.jp/) to the existing reference to close the gaps (https://github.com/gf777/T2T-zebra-finch/tree/main/patching). The model sequence was trimmed to include as much as possible of the sequence directly resolved by Verkko. To visualize read based evaluation, we first aligned HiFi and ONT reads to the assembly using minimap2 with the parameters `-ax map-hifi` and `-ax lr:hq` respectively. We then removed unmapped, secondary and

supplementary alignments (`-F 2308`) for HiFi and standard ONT reads, and unmapped and secondary alignments (`-F 260`) for ultra-long ONT reads using SAMtools v1.22. Next, we ran NucFlag v1.0.0a2[37,55] by setting the window size to 50kbp for the entire chromosome, and keeping the default 5kbp for the local tangle regions. The same NucFlag workflow was applied to assess the patched regions generated by the DEGAP software. ModDotPlot[41] was used to compute self-similarity dotplots of the patched regions with the commands `moddotplot static -f chrZ.fasta --region "chrZ:24000000-27500000" --axes-ticks 24000000 25000000 26000000 27000000 -o region1` and `moddotplot static -f chrZ.fasta --region "chrZ:67000000-69500000" --axes-ticks 67000000 68000000 69000000 -o region2`.

Gene models identification

To identify gene models on the resolved chromosome Z, EGAPx annotation from the T2T zebra finch assembly was transferred to the gap-resolved chromosome Z assembly using Liftoff.[36] Genomic coordinates corresponding to the *PAK3L* cluster boundaries were defined on the resolved chromosome Z. The Liftoff-transferred annotation was intersected with these intervals to extract all gene models located within each cluster. Gene models were then screened for duplication within the cluster boundaries by identifying repeated annotations of the same gene (or gene family). Each identified protein sequence was aligned against the reference genome using miniprot.[40] To simplify miniprot mapping results, we collapsed redundant alignments into a single representative call per genomic locus using an in-house script. Functional domains of the predicted proteins were identified using eggNOG-mapper v.5.0.2.[42] The specific analysis of *PAK3L* genes is documented here: https://github.com/gf777/T2T-zebra-finch/tree/main/chrZ/PAK3L.

DEGAP analysis

DEGAP requires a left and right boundary for each gap. To match the input for TTT, we aligned the final resolved assembly to the gapped sequence and trimmed to the last aligned base before and after each gap. DEGAP v2.0 (commit 2f4c318105c77b4c72efa4-d104156547977a348c) was used for all experiments. Initial runs force-joined gap2 without any inserted sequence due to a detected overlap in the boundary regions. We therefore disabled this initial merging and allowed DEGAP to use extension to fill the gap. All HiFi and ONT reads assigned to a node in the chr Z connected component by the verkko assembly were extracted. DEGAP was run with the command `DEGAP.py --mode gapfiller --seqleft $gapLeft --seqright $gapRight --hifi hifi.fastq.gz --ont ont.fastq.gz --flag left -t $cores --out $lPrefix` where `$gapLeft` was the sequence to the left of the gap and `$gapRight` was the sequencing following the gap. The `$gapRight` for gap 1 was equivalent to `$gapLeft` for gap2.

Cell Genomics, Volume *6*

Supplemental information

Automatic generation of model sequences

for complex regions in assembly graphs with TTT

Dmitry Antipov, Ying Chen, Marco Sollitto, Adam M. Phillippy, Giulio Formenti, and Sergey Koren

Methods S1. Related to STAR Methods.
Described methods assume that only one of *edge* and *reverse_complement(edge)* can be a part of the tangle. This is not true in the general case - inverted repeats may cause both *edge* and *reverse_complement(edge)* to be a part of the same traversal.
To address this issue, following changes should be made to described methods:

a) We should distinguish the multiplicity of an edge and its reverse complement.
Thus, for each edge in the assembly graph, we should have separate integer variables *mult(e)* and *mult(reverse_complement(e)).* Thus, to fix the multiple integer programming problem formulation, in condition 1 the vertex and its reverse complement should be considered separately and in conditions (2-5) *mult(e)* should be replaced by *(mult(e) + mult(reverse_complement(e)))*
b) Now the graph induced by the multiplicities can have more than one connected component. Because of the condition on the incoming and outgoing edges, there should be eulerian cycles through each of the components except one which contains boundary edges. Let's name those components as *cyclic components*, and the component with boundary edges as a *path component*. We can switch the orientation (replace all edges with their reverse complements) for all cyclic components without violating any of the conditions A1-A4 and changing the final score defined in A5. At least one of those new cyclic paths should be adjacent to some of the edges in the path component (otherwise the initial tangle would be disconnected), and thus the path component is extended. This can be repeated iteratively until no cyclic component remains.
c) Now we should allow not only reordering but also changing the orientation of the edges in the path. To do so, we define two additional operations: *inversion* and *reverse complement swap.*
   i) Inversion: Replace a subpath P between vertex A and its reverse complement vertex A' to its reverse complement P'
   ii) Reverse-complement swap: For two nonoverlapping intervals *(A, B)* and *(reverse_complement(B), reverse_complement(A))* between reverse-complement vertices in reverse order, reverse-complement and swap corresponding subpaths.

Those two operations can change multiplicity of an edge *e*, but preserve *(mult(e) + mult(reverse_complement(e)))*

To process tangles with two traversing haplotype paths (from S_1 to T_1 and from S_2 to T_2) after determining edge multiplicities we add auxiliary edge *aux* of multiplicity 1 from the end of first traversal T_1 to start of the second path S_2. Then, the final path is split in two by removing *aux* from it. To preserve pairing of incoming/outgoing edges, during optimization step C we forbid inversion of intervals containing *aux.* Additionally, in two haplotype cases we add a small penalty for each node of multiplicity > 1 that is present in only one of the haplotypes. This tweak is motivated by the observation that for diploid tangles it is unlikely to have a completely unbalanced traversal where all repeat units are assigned to the same haplotype.

Lemma 1: An Eulerian path in directed graph G can be transformed into any other Eulerian path in the same graph (with same start and end) through a sequence of swaps.

Let's proceed by contradiction. Assume there exist at least two distinct Eulerian paths from a starting vertex *S* to a terminal vertex *T* in a graph *G*. Let P_1 and P_2 be two such paths, with the premise that P_1 cannot be transformed into P_2 through any sequence of path swaps.

Let Ω be the set of all paths that can be generated by applying a series of swaps to P_1. From this set Ω, let's select a path, which we'll call P', that has the longest possible common prefix with P_2. Our goal is to show that if P' is not identical to P_2, we can always perform a swap on P' to create a new path, P'', which has an even longer common prefix with P_2. This will contradict the initial choice of P' as the path with the maximal common prefix.

Let the maximal common prefix of P' and P_2 be denoted by P_{prefix}, which terminates at a vertex *X*. Let the first edge after vertex *X* in path P' be *a*, and in path P_2 be *b*. By the definition of a maximal prefix, it must be that $a \neq b$.

Let us now consider a subgraph, G_r, formed by removing all edges of P_{prefix} from the original graph *G*. The remaining portions of the paths, P'_r (from P') and P_{2r} (from P_2), are both Eulerian paths within G_r , each starting at vertex *X* and ending at vertex *T*. The path P'_r begins with edge *a*, while P_{2r} begins with edge *b*. Since P'_r is an Eulerian path in G_r, it must traverse every edge in G_r , including edge *b*. Because P_{2r} starts at *X* with edge *b*, the source of edge *b* must be *X*.

Now our goal is to find a swap that would transform P'_r to start with b. If we find it, the same swap would increase the common prefix length between P' and P_2 which contradicts the choice of P'. Therefore, we can decompose the path P'_r into three distinct segments:

1. A cycle, $\mathbf{P'_c}$, which starts at *X* with edge *a* and eventually returns to *X*.
2. The single edge, *b* which connects *X* to a vertex we'll call *Z*.
3. A suffix path, $\mathbf{P'_s}$, which runs from vertex *Z* to the terminal vertex *T*.

Now, we will show that the vertex sets of P'_c and P'_s must be disjoint. Suppose they are not. This means there exists some vertex *Y* that is common to both the cycle P'_c and the path P'_s. Then we could then perform a swap of X-Y fragments in P'_c and $(b \to P'_s)$, and the resulting path would start from b. Let V'_c be the set of vertices in P'_c, and let V'_s be the set of vertices in P'_s. We have established that $V'_c \cap V'_s = \varnothing$.

Finally, consider the path P_{2r}. It starts from *X* with edge *b* and, as an Eulerian path, must traverse all edges of G_r, which includes the edge a starting from vertex X. This implies there must be a path from $X \in V'_s$ to $X \in V'_c$. Consequently, there must exist an edge, let's call it $e = (A, B)$, such that its source vertex $A \in V'_s$ and its destination vertex $B \in V'_c$.

Where could this edge *e* possibly lie in the path $P'_r = P'_c \to b \to P'_s$?

- It cannot be in P'_c, because its source vertex *A* is not in V'_c.
- It cannot be in P'_s, because its destination vertex *B* is not in V'_s.
- It's not *b* since destination of *b* is in V'_s

The edge *e* exists in the graph G_r but is not traversed by the path P'_r. This is a terminal contradiction, as it violates the premise that P' is an Eulerian path. Thus, our initial assumption must be false and any Eulerian path can be transformed into any other through a series of swaps.

Methods S2. Related to STAR Methods.
Precisely extracting tangles from an assembly graph is a complex question out of scope of this work. To minimize arbitrary decisions, we designed the following process for tangle identification based on the input assembly graph

1. Remove all bubbles (defined as an edge pair that shares incoming and outgoing vertices and have similar lengths - $l_1 < l_2 * 1.5; \ l_2 < l_1 * 1.5$)
2. Remove all tips (defined as edges with either no outgoing or incoming connections and shorter than 30kbp)
3. Compress all nonbranching paths
4. Repeat steps 1-3 until no further simplification is possible. Remove all edges that are longer than 1 Mbp
6. Consider all connected components in the remaining graph as tangle candidates
7. For each tangle candidate iteratively restore all adjacent edges that were removed in steps 1-4
8. Consider all edges adjacent to the tangle (in the initial graph) as boundary edges. If a boundary edge has more than one adjacent incoming(outgoing) edge and those edges are not in the tangle - add that boundary edge to the tangle and consider those incomings/outgoings as boundaries.

The aim of this pipeline is to get rid of large but trivial to resolve haploid and diploid regions and to reduce the regions in consideration as much as possible. Although TTT can be run on large tangles, an underlying assumption of coverage uniformity across the tangle is often violated for multimegabase-scale regions.

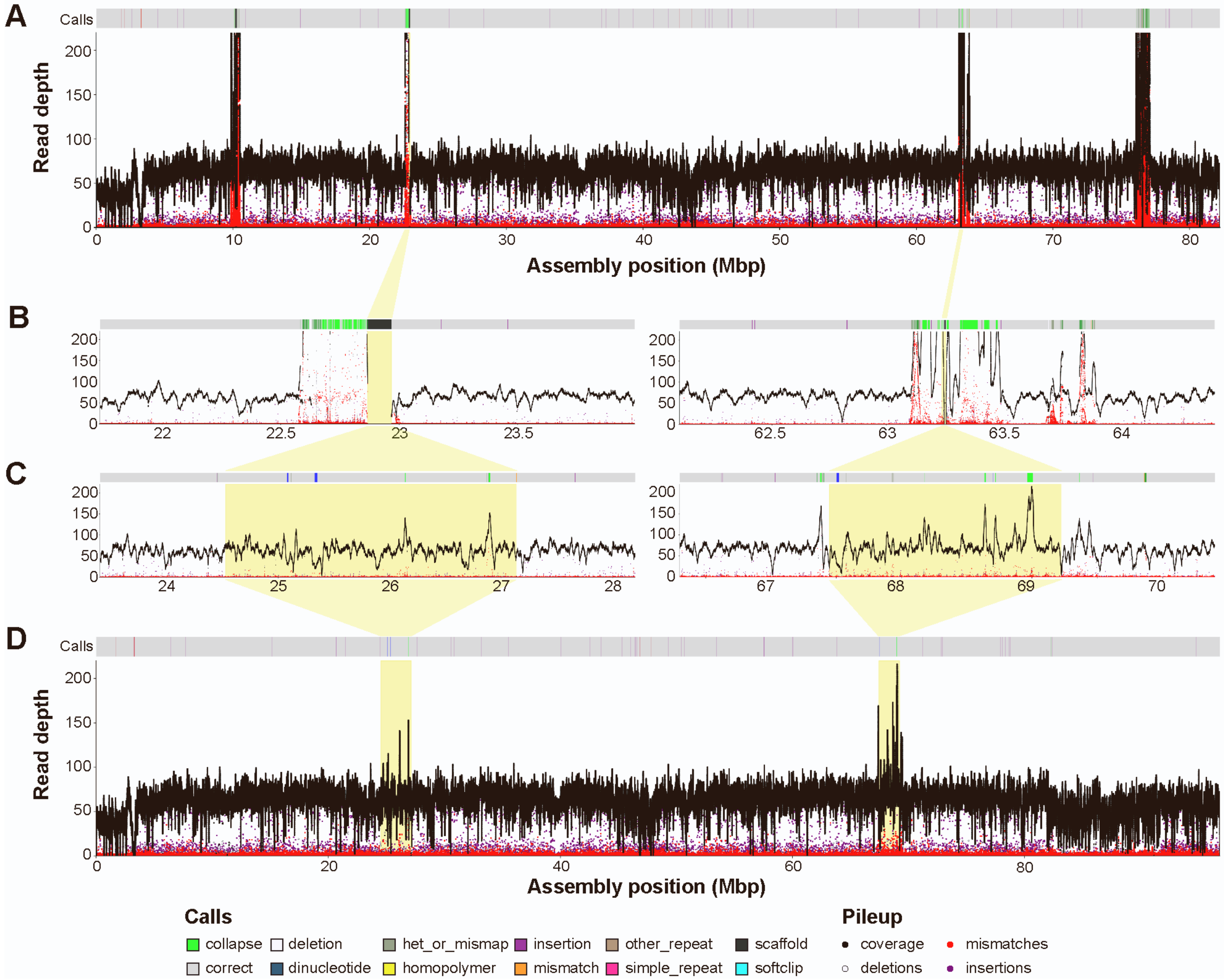

Figure S1. Nucflag HiFi coverage plots. Related to Figure 2. (A) zebra finch Chromosome Z before resolving the gaps (NCBI accession NC_133063.1), two tangle regions (B) before and (C) after running TTT, and (D) zebra finch Chromosome Z after resolving the four gaps. The start and end position for the plotting region in (B) and (C) are the same for each tangle, with the tangles highlighted in yellow. The total collapsed regions decreased from 124,253bp to 19,908bp in tangle 1 and from 147,035bp to 71,869bp in tangle 2.

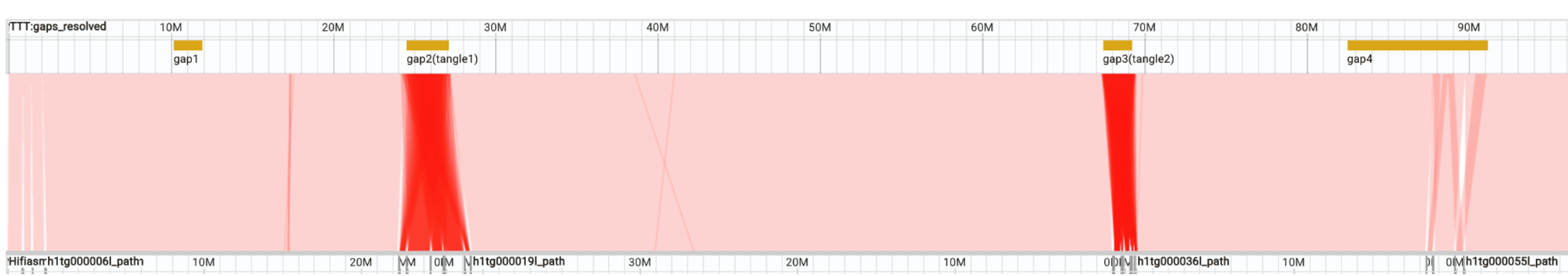

Figure S2. JBrowse2 synteny view. Related to Figure 2. Synteny between the gap-resolved Chr Z (top) and the Hifiasm assembly (bottom) from the same HiFi and ONT input data as the Verkko assembly. The first gap was resolved in Hifiasm while the other three gaps were not. There are 7 contigs aligning with the second gap (tangle 1), 6 contigs to the third gap (tangle 2) and 3 contigs to the fourth gap.

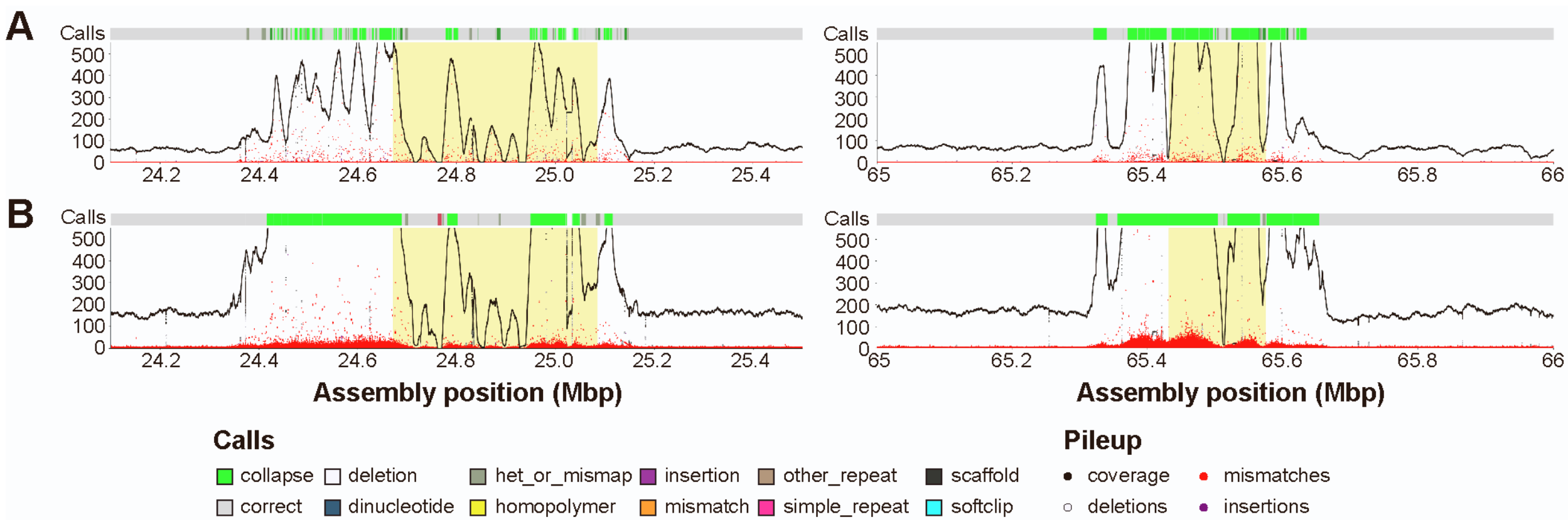

Figure S3. Nucflag plots of DEGAP resolutions. Related to Figure 2. (A) HiFi reads and (B) ONT reads for two tangle regions patched using DEGAP. Left is tangle 1 and right is tangle 2. The two tangle regions remain unresolved, with collapsed regions shown in green.

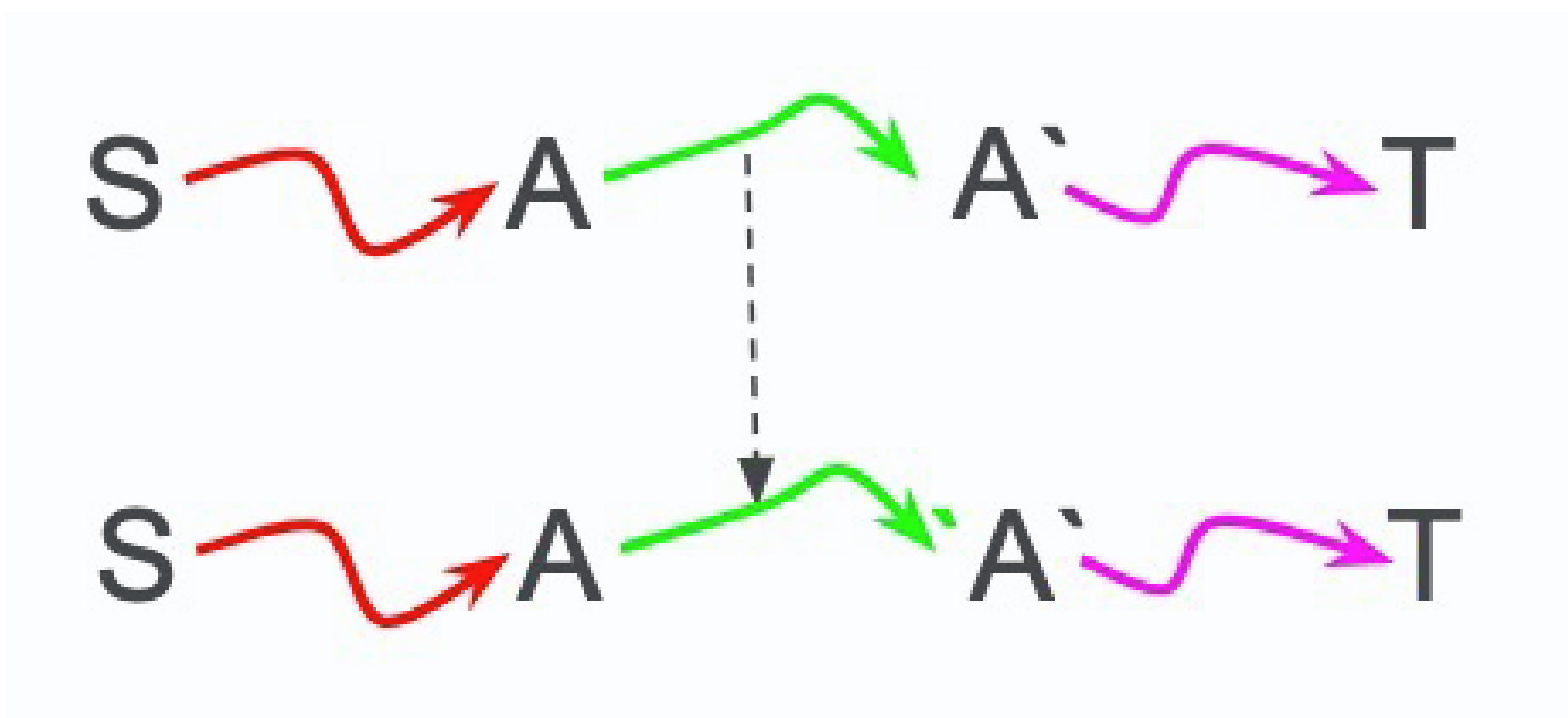

Figure S4. Inversion operation. Related to STAR Methods. Capital letters represent the vertices of the graph. Reverse-complement vertices and paths are shown with the symbol `. Inversion operation replaced the blue subpath with its reverse-complement.

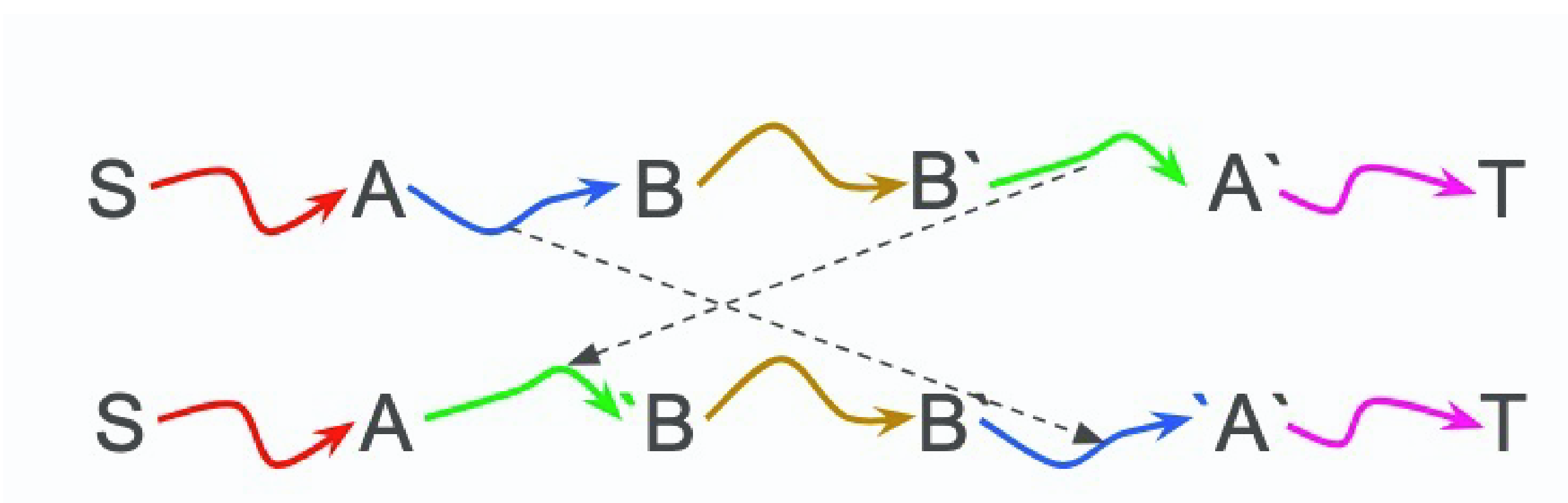

Figure S5. Reverse-complement swap operation. Related to STAR Methods. Capital letters represent the vertices of the graph. Reverse-complement vertices and paths are shown with the symbol `. Blue and green subpaths are replaced by their reverse-complements and swapped.

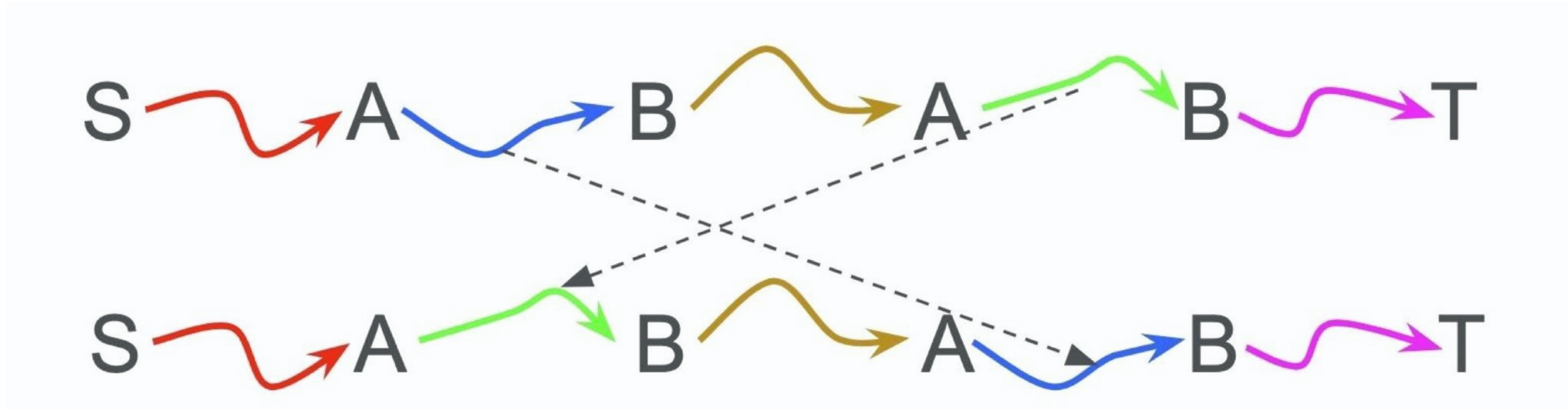

Figure S6. Swap operation. Related to STAR Methods. Capital letters represent the *vertices* of the graph. Colored arrows represent different subpaths in the assembly graph. A swap operation rearranges the green and blue subpaths.

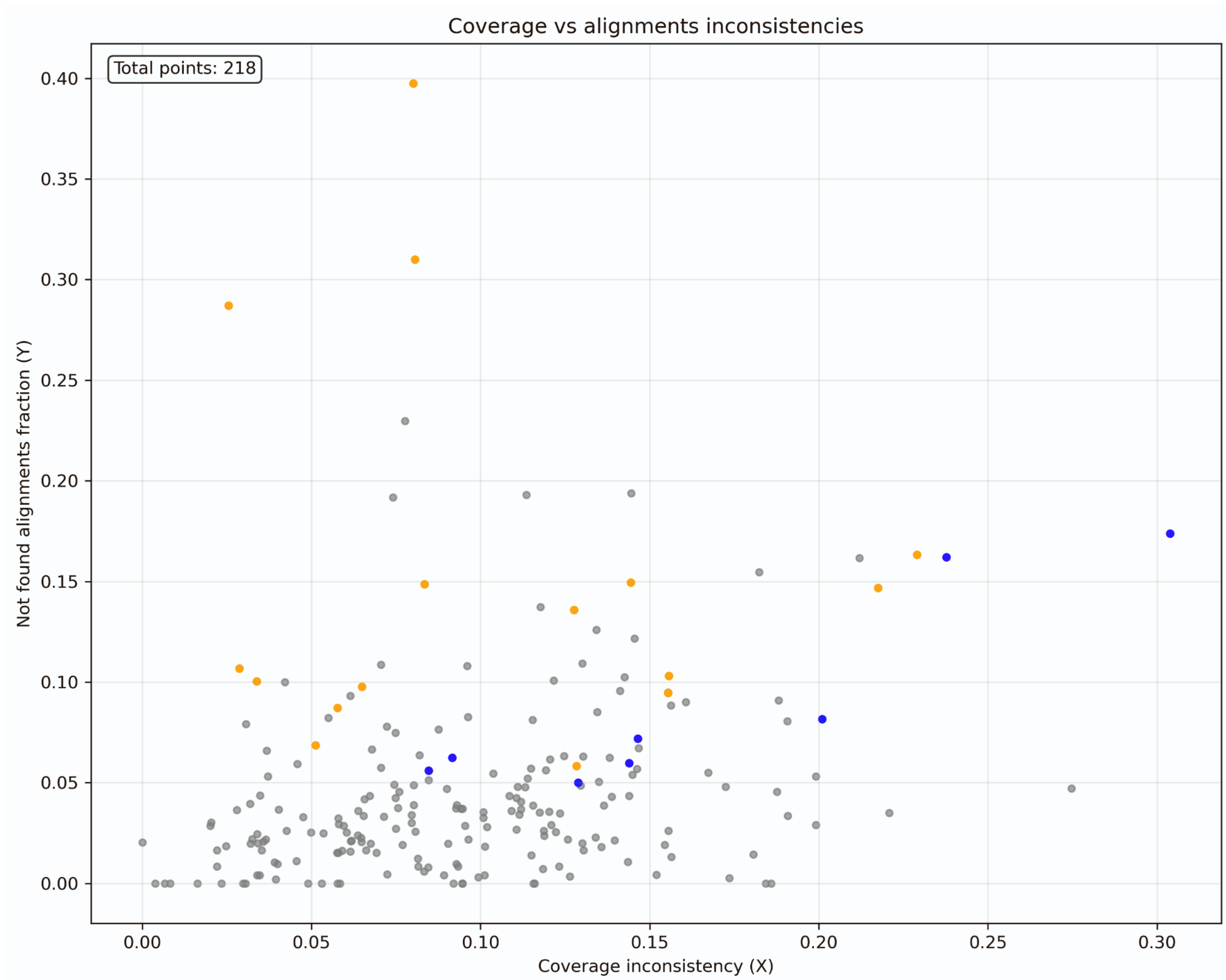

Figure S7. Internal TTT evaluation on tangles of the HG002 hifi-only assembly. Related to STAR Methods. Orange points represent the tangles where verkko's resolution is better than TTT's and blue points represent the tangles where TTT provides better resolution.

Table S1. Details on tangles in the T2T-HG002 datasets where TTT and Verkko provided different traversals. Related to Figure 1. score_diff is the difference between minimap2 scores of the best alignments of TTT and Verkko traversals of corresponding regions to the HG002 reference. Broken alignment column represent cases where at least one of Verkko and TTT was not aligned to the reference in one block.

tangle	verkko_chr	verkko_start	verkko_end	score_diff	broken alignment	diploid tangle
tangle_64_0	chr4_PATERNAL	34374570	35640222	0	FALSE	TRUE
tangle_64_1	chr4_MATERNAL	34404725	37634837	-69045	FALSE	TRUE
tangle_0_0	chrY_PATERNAL	13824350	16541148	3599133	TRUE	FALSE
tangle_181_0	chr11_PATERNAL	35474010	37872656	-1176	FALSE	TRUE
tangle_181_1	chr11_MATERNAL	35464846	38707935	0	FALSE	TRUE
tangle_210_0	chr16_PATERNAL	28739539	32048130	96770	FALSE	FALSE
tangle_81_0	chr19_PATERNAL	12727459	12824748	0	FALSE	TRUE
tangle_81_1	chr19_MATERNAL	12712630	12809901	-43	FALSE	TRUE
tangle_97_0	chr4_MATERNAL	41427476	41549214	-20	FALSE	TRUE
tangle_97_1	chr4_MATERNAL	41427545	41549214	0	FALSE	TRUE
tangle_193_0	chr6_PATERNAL	36459925	37216904	0	FALSE	TRUE
tangle_193_1	chr6_MATERNAL	36401014	37158213	-20	FALSE	TRUE
tangle_224_0	chr1_PATERNAL	115850076	115983245	-20812	FALSE	TRUE
tangle_224_1	chr1_MATERNAL	109908159	110004602	0	FALSE	TRUE
tangle_155_0	chr5_PATERNAL	64946203	66230825	0	FALSE	TRUE
tangle_155_1	chr5_MATERNAL	60945245	62230195	4	FALSE	TRUE
tangle_220_0	chr1_MATERNAL	90733718	93987681	-42	FALSE	FALSE
tangle_3_0	chr17_PATERNAL	16395042	19880444	-1049401	TRUE	TRUE
tangle_3_1	chr17_MATERNAL	16424231	19285942	0	FALSE	TRUE
tangle_58_0	chrY_PATERNAL	38094517	42080898	2222986	TRUE	FALSE
tangle_146_0	chr9_MATERNAL	81605645	81698051	-7492	FALSE	TRUE
tangle_146_1	chr9_PATERNAL	73565313	73646472	0	FALSE	TRUE
tangle_216_0	chrY_PATERNAL	24070694	29418231	-3014931	TRUE	TRUE

tangle_216_1	chrY_PATERNAL	29413938	32962405	-2439096	TRUE	TRUE
tangle_123_0	chr4_PATERNAL	112053555	112153686	42	FALSE	TRUE
tangle_123_1	chr4_MATERNAL	111462950	111563036	0	FALSE	TRUE
tangle_7_0	chr9_MATERNAL	33724352	37810563	-30745	FALSE	TRUE
tangle_7_1	chr9_PATERNAL	32289742	34760836	0	FALSE	TRUE
tangle_56_0	chr16_PATERNAL	44686575	46132003	31	FALSE	TRUE
tangle_56_1	chr16_MATERNAL	42295876	43741863	0	FALSE	TRUE
tangle_78_0	chr1_PATERNAL	72215243	73688345	0	FALSE	TRUE
tangle_78_1	chr1_MATERNAL	72156885	73630883	-20	FALSE	TRUE
tangle_60_0	chr19_PATERNAL	18261080	20670064	-295425	FALSE	FALSE
tangle_235_0	chr10_PATERNAL	27412257	30621726	-31528	FALSE	FALSE
tangle_77_0	chr7_MATERNAL	89009753	89089787	-43	FALSE	TRUE
tangle_77_1	chr7_PATERNAL	88289429	88369464	0	FALSE	TRUE
tangle_75_0	chr19_MATERNAL	26290203	28691624	-7298	FALSE	TRUE
tangle_75_1	chr19_PATERNAL	26704211	28763530	0	FALSE	TRUE
tangle_140_0	chr10_PATERNAL	14624063	14878108	0	FALSE	TRUE
tangle_140_1	chr10_MATERNAL	14615380	14869002	5	FALSE	TRUE
tangle_15_0	chr13_PATERNAL	33183516	33494280	45	FALSE	TRUE
tangle_15_1	chr13_MATERNAL	31932813	32243681	0	FALSE	TRUE

Table S2. Complexity of tangles in the T2T-HG002 dataset used for TTT benchmarking. Related to Figure 1. Tangles were identified using the assembly graph structure with extract_all_tangles.py script (included in TTT release, described in Methods S2). TTT-inapplicable tangles (tangles that had neither two nor four boundary nodes and tangles with four boundary nodes that were not resolved or scaffolded by Verkko) are not included. Note that a median multiplicity of 1 can occur when a tangle contains both conserved and diverged repeats. These diverged nodes, with multiplicity 1, commonly outnumber the conserved nodes.

T2T-HG002 tangles	max	mean	median
# of nodes (gfa)	4185	32.3	7
# max links per node (gfa)	5	2.65	2

multiplicities	19	1.3	1

Table S3. Complexity of tangles in the giraffe dataset used for TTT benchmarking. Related to Figure 1. Tangles were identified using the assembly graph structure and gaps in verkko-gapped assembly scaffolds. Only TTT-applicable (scaffolded, not multi-haplotype) tangles were taken into account.

TTT-resolved tangles	max	mean	median
# of nodes (gfa)	99	26.4	17
# max links per node (gfa)	5	2.7	2
multiplicities	24	2.1	1
TTT-resolved tangles also resolved in verkko-normal	max	mean	median
# of nodes (gfa)	46	12.3	9
# max links per node (gfa)	4	2.6	2
multiplicities	4	1.3	1

Table S4. Classification of gaps in 227 HPRC assemblies. Related to STAR methods. We consider boundary edge pairs as valid if a) they separate tangles from the rest of the graph b) they are presumably unique. This is estimated by requiring edge length to be larger than 40Kbp and edge coverage being relatively close to the median edge coverage for the dataset (0.5 * median_cov < cov < 1.5 * median_cov).

Category	Count	%
Valid: in a single haplotype tangle	3023	35.4%
Valid: in a two haplotype tangle	1152	13.5%
Invalid: gap in graph	2358	27.6%
Invalid: in tangle shared between >2 scaffolds	1538	18.0%
Invalid: unable to find unique boundary nodes	461	5.4%
